# In-Depth Molecular Dynamics Simulations Reveal Ligand-Induced Modulations of the HSPA8-SARS-CoV-2 Spike Protein Interaction

**DOI:** 10.3390/ijms27104288

**Published:** 2026-05-12

**Authors:** Liberty T. Navhaya, Mokgerwa Z. Monama, Thabe M. Matsebatlela, Xolani H. Makhoba

**Affiliations:** 1 Department of Biochemistry, Microbiology, and Biotechnology, University of Limpopo, Turfloop Campus, Sovenga 7270, South Africa; 202417284@myturf.ul.ac.za (L.T.N.); mokgerwa.monama@ul.ac.za (M.Z.M.); thabe.matsebatlela@ul.ac.za (T.M.M.); 2 Department of Life and Consumer Sciences, College of Agriculture and Environmental Sciences, University of South Africa (UNISA), Florida Campus, Roodepoort 1709, South Africa

**Keywords:** heat shock protein, HSPA8, molecular dynamics simulations, SARS-CoV-2 spike protein

## Abstract

Coronavirus disease 2019 continues to pose global health challenges, with the pandemic significantly burdening several economies, healthcare systems, and the social lives of individuals. Furthermore, new cases continue to be reported, underscoring the need for therapeutic strategies targeting conserved regions and host–virus interactions. Building on earlier virtual screening for small molecules, all-atom molecular dynamics simulations and binding-free-energy calculations were performed to elucidate how the two previously identified small molecules (NSC36398 and NSC281245) may affect the dynamic behaviour of the interaction between heat shock 70 kDa protein 8 (HSPA8) and the severe acute respiratory syndrome coronavirus 2 (SARS-CoV-2) spike glycoprotein. Post-MD analyses refined prior docking predictions, where NSC281245 was found to bind tightly to the complex with limited perturbations at the HSPA8-spike protein interaction surface, whereas NSC36398 appeared to induce allosteric-like domain-level destabilisation effects while maintaining stable polar contacts with the protein. Our findings demonstrate the potential of NSC36398 as a promising modulator for disrupting the HSPA8-spike protein complex, which may serve as a structural lead for designing next-generation inhibitors of host–virus interactions.

## 1. Introduction

Coronavirus disease 2019 (COVID-19) is a term coined by different scientists to describe the new strain of coronavirus that belongs to the *Coronaviridae* family. This virus emerged in Wuhan, China, in December 2019 [1]. Coronaviruses are known to infect humans and animals, causing respiratory illness ranging from a mild common cold to fatal respiratory failure. It has been depicted that the COVID-19 virus spreads faster than its predecessors, namely, the Middle East Respiratory Syndrome Coronavirus (MERS-CoV) and Severe Acute Respiratory Syndrome Coronavirus (SARS-CoV). Classified as Severe Acute Respiratory Syndrome Coronavirus 2 (SARS-CoV-2), COVID-19 has been hypothesised to have originated from bats and then migrated to an intermediate host before transmitting to humans. As highlighted, this resulted in pneumonia similar to that caused by the preceding coronaviruses [1].

Structurally, SARS-CoV-2 comprises a viral envelope (E) containing a lipid bilayer with embedded club-shaped spike (S) glycoproteins protruding from the viral surface, including the membrane (M) proteins [2]. The SARS-CoV-2 spike protein has been a key target for drug design and therapeutic development as it plays a crucial role in the viral life cycle. The spike protein primarily binds to the host cell receptor, the angiotensin-converting enzyme 2 (ACE2), facilitating viral entry into the host cell. A deeper look into the SARS-CoV-2 spike protein indicated that it consists of a short intracellular C-terminal segment, a transmembrane (TM) domain, and an extracellular N-terminus [3]. Additionally, the spike protein exists as a metastable prefusion conformation; when the viral protein interacts with host cell receptors, it undergoes structural rearrangements, allowing the virus to fuse with the host cell membrane. The spike protein is cleaved into S1 and S2 subunits, allowing the spike’s S1 subunit, which has the receptor-binding domain (RBD), to interact and bind with the ACE2 receptor, mediating viral entry [4].

SARS-CoV-2 is an obligatory pathogen that lacks the necessary components for replication within the host, relying instead on the host biomolecules for its survival and propagation. Among the most targeted host factors are heat shock proteins (HSPs) that play pivotal roles in maintaining cell homeostasis, protecting cells from harsh conditions, assisting in protein folding, and presenting immune and inflammatory compounds [5]. HSPs are classified according to molecular weight, with heat shock protein 70 (HSP70) being one of the most extensively studied molecular chaperones. Its constitutively expressed counterparts, known as heat shock cognates, perform similar functions in the absence of stress. For instance, heat shock cognate 71 kDa protein (HSC70), also known as heat shock 70 kDa protein 8 (HSPA8), plays vital roles in regulating protein translocation, protein quality control, and the refolding of misfolded proteins [6,7].

**Figure 1:**
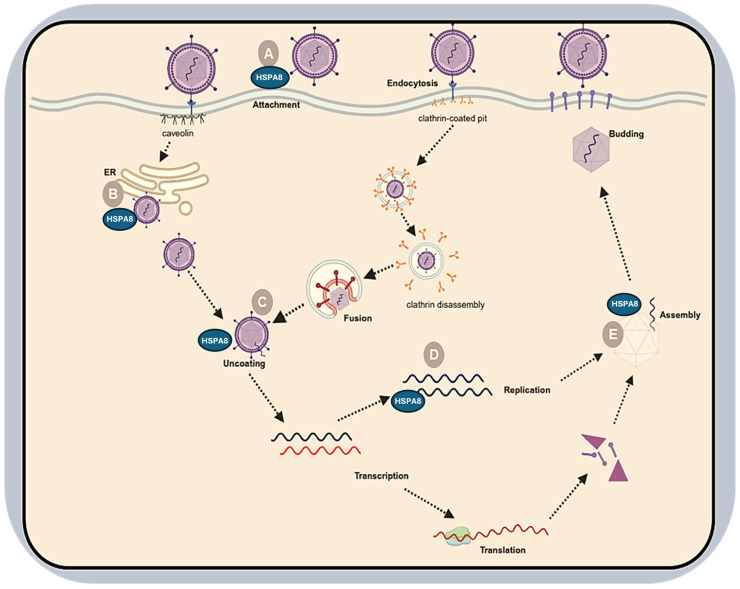
Multifunctional roles of HSPA8 during viral infection. HSPA8 participates at multiple stages of the viral life cycle: (A) at the cell surface to assist viral entry, (B) during uncoating (e.g., clathrin removal and ATP-dependent capsid disassembly), (C) by interacting with viral proteins/genomes to modulate replication, (D) during virion morphogenesis, and (E) by promoting viral morphogenesis by interacting with capsid proteins. Arrows indicate reported positive or negative regulation. Here, we listed the viruses reported to hijack HSPA8 at each stage: (A) Rotavirus A (rotavirus) and Dengue virus (DENV) [9,10,11]; (B) Simian virus 40 (SV40) [11]; (C) Rotavirus A (rotavirus) [11,12]; (D) Dengue virus (DENV), Murine gammaherpesvirus 68 (MHV68), Rabies lyssavirus (RABV), Hepatitis B virus (HBV), Ebola virus (EBOV), Duck hepatitis B virus (DHBV), and Enterovirus A71 (EV-A71) [10,11]; (E) Hepatitis C virus (HCV), Human papillomavirus (HPV), and Hepatitis B virus (HBV) [11]. Created in BioRender. Navhaya, L.T. (2025) https://app.biorender.com/illustrations/693ffc90402170b21d541096?slideId=987182b1-1902-4e86-9668-9487ea5b921d (accessed 27 October 2025).

Numerous studies have established HSPA8 as one of the key molecular chaperones hijacked and manipulated by viruses at various stages of the viral life cycle, including attachment, internalisation, uncoating, and intracellular trafficking (Figure 1). Importantly, recent work has shown that HSP70 can interact with SARS-CoV-2 entry machinery. In fact, Joshi et al. (2024) [8] provided direct, cell-based evidence that members of the HSP70 family engage the SARS-CoV-2 RBD. The study demonstrated that biochemical and cellular data showed that inducible HSP70 binds to both human ACE2 and the viral RBD. The study further indicated that pharmacological inhibition of the inducible HSP70 with a phenylethynesulfonamide derivative, PES-Cl, significantly reduced spike RBD-mediated viral entry and overall replication. These findings suggest that HSP70 family members can modulate SARS-CoV-2 infectivity, thereby identifying HSP70 inhibition as a potential antiviral strategy [9]. Building on the conclusions by Joshi et al. (2024) [8], we hypothesise that HSP70 family members contribute to SARS-CoV-2 entry and post-entry events, and that different paralogs play distinct roles in the viral life cycle, depending on their expression and localisation. In particular, we propose that HSPA8 potentially influences SARS-CoV-2 infection by acting as either an attachment or post-attachment factor, facilitating viral entry, or by participating in clathrin-mediated endocytosis and uncoating, thereby facilitating viral RNA release (Figure 1).

**Figure 2:**
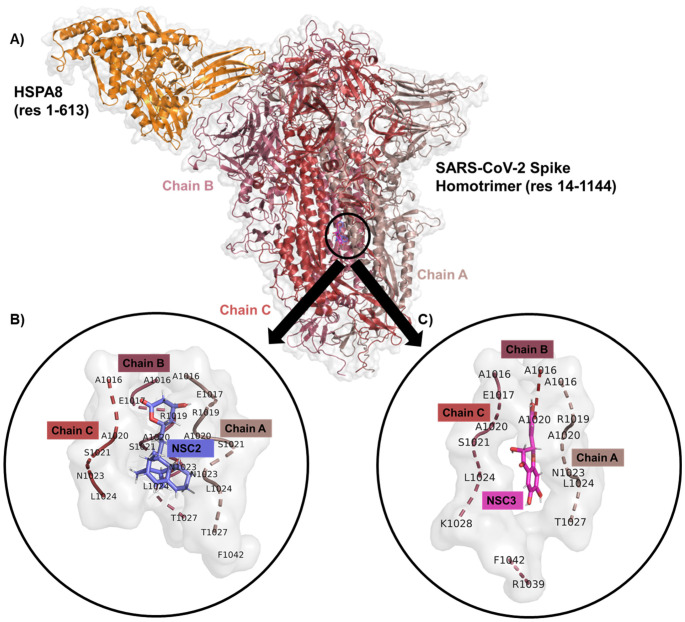
Predicted binding poses of the (**A**) HSPA8-spike protein complex, two naturally occurring compounds, (**B**) NSC281245 and (**C**) NSC36398, identified through virtual screening, ADMET analysis, and Prime MM/GBSA calculations.

Numerous experimental studies in other virus families provide further support for the role of HSPA8 in surface-associated endocytosis and uncoating, as well as in viral infection overall [9,10,11,12,13,14,15,16,17]. Informed by this, in our previous work, we attempted to predict the extensive possible interactions between the SARS-CoV-2 spike glycoprotein and the HSPA8 molecular chaperone, achieving a stable complex (HSPA8-spike protein complex; Figure 2) [18,19]. Based on our findings, the host HSPA8 substrate-binding domain (SBD see Supplementary File S1, Figure S1A) may interact with the SARS-CoV-2 RBD from chain A and chain B (see Supplementary File S1, Figure S1B), forming extensive hydrogen bonds and a single salt bridge with a bond distance ranging from 1.6 to 2.5 Å (Figure S2) [18,19]. The depicted interactions were determined to be pervasive, meaning that more energy is required to break these bonds, indicating a stably docked protein–protein complex.

Several well-characterised reagents and small molecules have been reported to engage HSP70 family members or the SARS-CoV-2 spike glycoprotein and can be used as positive controls in biochemical assays. Examples include HSP70 inhibitors such as VER-155008 (an ATP-competitive NBD binder) and JG-98 (an allosteric inhibitor) [20,21]. Phenylethynesulfonamide derivatives (PES/PES-Cl) were recently reported to reduce SARS-CoV-2 entry and replication, as previously reported [8]. Several small-molecule RBD/ACE2 disruptors emerged from high-throughput and structure-based screening studies. Established spike-targeting controls include neutralising monoclonal antibodies, coronavirus fusion peptides (lipo-conjugates), and heparin-sulphate mimetics [22,23]. To our knowledge, however, no experimentally validated naturally occurring small-molecule compounds have been shown to selectively target the HSPA8-SARS-CoV-2 Spike Protein (HSPA8-spike protein) complex. The available HSP70 inhibitors target the chaperone broadly and thus act as indirect controls. Importantly, our study intentionally focused on naturally occurring compounds from the literature as starting scaffolds, often having different modes of action and physicochemical properties compared with the biologics and synthetic inhibitors mentioned above. This difference limits direct comparison with the present work and underscores a gap in the literature.

We therefore continue our work wherein we previously applied ADMET analysis, molecular docking, and Prime Molecular Mechanics with Generalised Born and Surface Area solvation (MM/GBSA), and identified two naturally occurring compounds, 2-(3,4-Dihydroxyphenyl)-3,6,7-trihydroxy-2,3-dihydro-4H-chromen-4-one (NSC36398) and mevastatin (NSC281245) as promising inhibitors for the protein complex (Figure 2; Table 1) [19,24]. Key residues (positions 1014−1023 and 1035−1068) forming part of the S2 central helix and β-hairpin were determined as the residues involved in interactions with NSC36398 and NSC281245. These regions have been reported to exhibit high structural and sequence conservation among β-coronaviruses, indicating that small-molecule binders targeting these regions may retain their inhibitory activities more effectively against future variants [19].

In this study, all-atom molecular dynamics (MD) simulations of the wild-type ligand-free system (apo state) of the HSPA8-spike protein complex and the ligand-bound system (HSPA8-spike-NSC36398 complex and HSPA8-spike-NSC281245 complex) were performed to investigate the dynamics of the HSPA8-spike and HSPA8-spike-ligand complexes as a function of time. Post-MD analyses focused on structural analysis, conformational dynamics analysis, interaction analysis, solvation exposure analysis, and binding free energy calculations.

## 2. Results and Discussion

Several recent studies demonstrate that focused MD on pre-formed complexes, often following docking studies, is an accepted and powerful strategy for interrogating how ligand binding modulates protein–protein interfaces and downstream domain dynamics [25,26,27]. Studies comparing apo and holo conformations have shown that ligand binding can drive distinct conformational transitions that are detectable on timescales accessible to well-replicated MD simulations, and such apo/holo comparisons are widely used to interpret ligand-specific effects [28]. Specific to the HSP70 family, recent MD investigations revealed that chaperone allostery and ligand-linked dynamics are tractable targets for computational analysis and provide insightful mechanistic hypotheses that can be validated experimentally [29]. We therefore adapted a similar interface-focused study, using the previously identified HSPA8-spike protein complex in both ligand-free and ligand-bound systems to investigate the effects of ligand binding.

### 2.1. Global Perturbation Effects of NSC36398 and NSC281245 on the SARS-CoV-2 Spike-HSPA8 Complex

**Figure 3:**
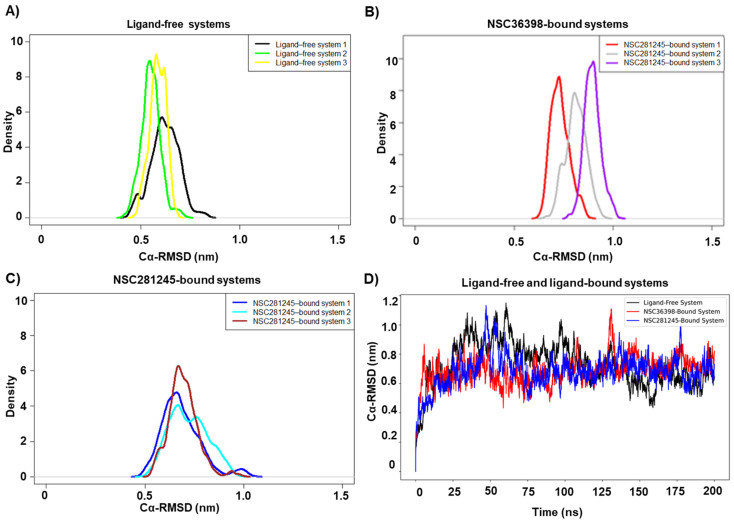
KDE plots for (**A**) Ligand-free system, (**B**) NSC36398-bound system, and (**C**) NSC281245-bound systems. (**D**) The captured Cα-RMSD plots of the ligand-free system, NSC36398-bound system, and NSC281245-bound system (with reference to their initial frames), generated using gmx rms of the full 200 ns trajectories, showing the initial increase in Cα-RMSD (0–100 ns) and the convergence phase of the MD simulations (100–200 ns).

Replicate kernel-density estimates (KDEs), verified reproducible ensembles, and captured the distinct conformational behaviour of each system (Figure 3A–C). The width of the KDE plots represents the amount of conformational sampling, while peaks represent highly sampled conformations. The KDE plots revealed distinct conformational behaviour in both ligand-free and ligand-bound systems. The ligand-free system (apo) displayed tightly clustered KDE peaks (≈0.48–0.70 nm; Figure 3A), signifying a stable conformational ensemble over time [30,31]. The ligand-bound systems displayed consistent, reproducible shifts towards higher RMSD values (NSC36398 ≈ 0.60–1.10 nm; NSC281245 ≈ 0.49–1.10 nm) (Figure 3B,C), signifying a modest increase in conformational flexibility upon ligand binding. Based on these replicate KDEs, a single, representative replicate from the ligand-free system was selected and set for formal convergence testing. It is also prudent to note that a single replicate for the ligand-free and ligand-bound systems was selected and used for all subsequent analyses (Figure 3D).

Based on the ligand-free system’s C-alpha (Cα) RMSD line plot, we initially suspected convergence around 100 ns (see Supplementary File S1, Figure S3A). To supplement our initial observation, block-averaging was used to decipher convergence with block sizes ranging from 1 to 200,000, through an ad hoc, in-house script. An optimal block size of ~100,000 was determined as shown by the distinct plateau (see Supplementary File S1, Figure S3B), relaying a standard error of mean (SEM) ~0.16 for an average Cα-RMSD of 0.73 nm, further supporting the initial convergence observation. Hence, to limit the SEM for the converged data, we proceeded to analyse the trajectories for the remaining 50 ns (henceforth referred to as the converged data/trajectory) for all subsequent analyses, unless otherwise stated. It is important to note that the Lilliefors test was initially applied to the converged trajectories for the generated RMSD, RMSF, and Rg datasets, confirming that the distributions were non-normal. Pairwise comparisons between the protein structures of the ligand-free system and ligand-bound systems using the Mann–Whitney U test, a non-parametric statistical approach, were therefore explored to determine whether the ligands showed evidence of significant structural effects (see Supplementary File S1, Table S1).

To begin, Cα-RMSD analysis of the apo system revealed that it exhibited relatively lower Cα-RMSD values compared to the ligand-bound systems (Figure 3D; see Supplementary File S1, Figure S4), consistent with compaction or higher structural stability (median ≈ 0.64 nm). The NSC281245-bound system, however, displayed a median Cα-RMSD (≈0.69 nm), consistent with a ligand stabilising the native fold without major perturbations. Conversely, NSC36398 binding displayed a modest increase in the median Cα-RMSD (≈0.72 nm), indicating a potential ligand-induced shift in the conformational ensemble rather than the observed preservation of the native state. These RMSD findings align with the KDE profiles, corroborating the simulations’ stability and reproducibility [30,31,32]. Additionally, significant differences in Cα-RMSD were found between the ligand-bound proteins and the ligand-free protein, which may further highlight the global perturbation effects of the NSC36398 and NSC281245 ligands. Following this global structural analysis, domain-specific RMSD analyses were conducted to investigate the local structural stability of key functional domains within the ligand-free and ligand-bound protein complex systems.

### 2.2. NSC36398’s Effects on the Local Stability of the HSPA8 Substrate-Binding Domain (SBD)

Domain-specific analyses revealed some ligand effects on the host HSPA8 substrate-binding domain (SBD, residues 349–509), known for recognising and binding to misfolded and unfolded proteins, acting as a chaperone [19,33].

**Figure 4:**
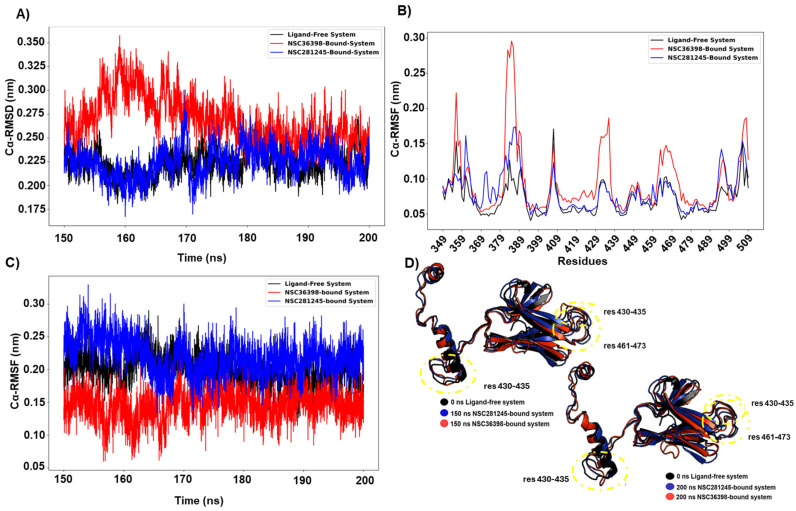
(**A**) Cα-RMSD and (**B**) Cα-RMSF line plots of the host HSPA8 SBD (residues 349–509) of the ligand-free system (black plot), NSC36398-bound system (red plot), and NSC281245-bound system (blue plot), estimated with reference to their initial structure. (**C**) Captured Rg of host HSPA8 SBD. (**D**) SBD structural comparison of the ligand-free reference (black; cartoon representation) at 0 ns, and the NSC36398-bound system (red; cartoon representation) and NSC281245-bound system (blue; cartoon representation) SBDs at 150 and 200 ns.

Figure 4A illustrates that the ligand-free system showed relatively lower structural deviations (apo system ≈ 0.22 nm) than that of the NSC36398-bound system and similar to that of the NSC281245-bound system, as indicated by the estimated Cα-RMSD values for the converged trajectory. Interestingly, the SBD of the NSC36398 system exhibited the highest median RMSD (≈0.26 nm) compared to the ligand-free and NSC281245-bound systems (both ≈ 0.22 nm). This suggests that NSC36398 significantly increases the distal SBD’s backbone mobility while affecting the local fluctuations of some residues, e.g., residue positions 379–390 that map the inter-domain linker, and 430–435 and 462–473 of the β5-β6 regions that contribute to co-chaperone interactions and structural stability [34,35,36,37]. This was further informed by the Cα-RMSF comparison to the apo system (Figure 4B,C), where NSC36398 showed elevated per-residue fluctuations (NSC36398 ≈ 0.08 nm versus apo ≈ 0.06). Additionally, NSC36398 binding exhibited a slightly lower median Rg value (2.18 nm; Figure 4D) compared with the apo (median Rg ≈ 2.20 nm), a pattern that may reflect a modest domain compaction occurring concomitantly with increased local and backbone mobility. NSC281245, however, appeared to yield mostly negligible SBD dynamic changes across the tested metrics when compared to the apo. Overall, these data indicate that NSC36398 binding is associated with increased SBD mobility and localised perturbations in regions important for substrate binding and stability.

### 2.3. NSC36398 and NSC281245’s Impact on the SARS-CoV-2 Chain A and B Spike Receptor-Binding Domain (RBD)

**Figure 5:**
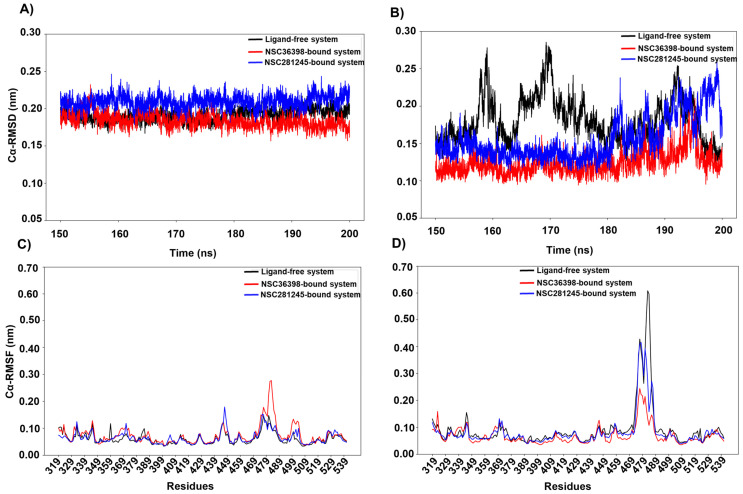
Captured Cα-RMSD plots for (**A**) chain A receptor-binding domain (residues 319–541), (**B**) chain B receptor-binding domain (residues 319–541), and Cα-RMSF plot (**C**) for chain A receptor-binding domain, (**D**) chain B receptor-binding domain.

Due to the critical function and direct interaction of chain A and B receptor binding domain (RBD) (residues 319–541) and receptor binding motif (RBM) (residues 437–508) with the host HSPA8 SBD [18,19], respectively, both the RBD and RBM were investigated in the ligand-free and ligand-bound systems on a per-chain basis (Figure 5). Additionally, the dynamics of the HSPA8-spike interface were also investigated. As mentioned previously, the RBD is a functional domain responsible for initiating crucial interactions that drive viral attachment to host cell receptors, thereby initiating viral entry [19,33]. Hence, the spike’s chain A and B RBD, which formed intermolecular interactions with the HSPA8 SBD, warranted scrutiny.

Analyses of the chain A spike RBD (Figure 5A,C) indicated some perturbation effects due to ligand binding. Figure 5A shows that the NSC281245-bound system displayed the highest structural deviations (median Cα-RMSD ≈ 0.21) relative to the ligand-free (median Cα-RMSD ≈ 0.19 nm) and NSC36398-bound systems (median Cα-RMSD ≈ 0.18 nm) as indicated by the estimated Cα-RMSD values. Per-residue fluctuations assessed by Cα-RMSF (Figure 5C) for chain A RBD are broadly similar across all three systems (ligand-free median ≈ 0.06 nm; NSC36398 median ≈ 0.06 nm; NSC281245 median ≈ 0.06 nm). NSC36398 binding resulted in an increased localised residue fluctuations around residues 473–487 and 497–502 (see Supplementary File S1, Figure S5A), consistent with RBM perturbation and ACE2-antigenic loop destabilisation. Residue S477 (≈475–477 region) sits in the receptor-binding motif (RBM) and substitutions at S477 (for example, S477N) have been shown to enhance ACE2 binding. Changes to this region, therefore, directly modulate receptor affinity [38,39]. By contrast, the 481–485 stretch (centred on E484) forms a short loop in the RBM that contributes to the ACE2-contacting ridge and is a well-documented antigenic/escape hotspot; mutations at E484 reduce neutralisation by convalescent and vaccine sera, as well as many monoclonal antibodies [40,41]. NSC281245 binding induces a significant increase in global structural deviations but minimal RMSF changes, whereas NSC36398’s effect is more localised (Figure 5C; see Supplementary File S1, Table S1). The Rg analysis (see Supplementary File S1, Figure S6A) indicates a moderate yet significant compaction of the chain A RBD. Instead, the observed modest differences in RMSF traces (NSC36398-bound system) and the elevated RMSD (NSC281245-bound system) are consistent with more subtle, localised or distributed perturbations rather than large-scale structural shifts.

Due to spike-trimer asymmetry and possible differences in inter-chain exposure and contacts, the structural and conformational behaviour of chain B RBD during ligand binding may differ from that of chain A [42,43]. Figure 5B shows that the ligand-free chain B RBD exhibits substantially large and more frequent structural excursions than the ligand-bound systems (ligand-free median RMSD ≈ 0.17 nm; NSC36398 median RMSD ≈ 0.11 nm; NSC281245 ≈ 0.16 nm), as reflected in the Cα-RMSD time plots. The pronounced peaks in the ligand-free time plot are noticeably dampened in both ligand-bound systems, especially with NSC36398, indicating ligand-binding-associated stabilisation of the RBD. Cα-RMSF mirrors the observed structural perturbations, where ligand-free chain B RBD (median RMSF ≈ 0.08 nm; see Figure 5D) shows increased residue fluctuations around residues 470–487, which maps to the spike’s RBM [40]. Ligand binding at the S2 subunit dampened the increased residue fluctuations, with NSC36398 binding displaying the most reduced residue flexibility (NSC36398 median RMSF ≈ 0.06 nm; see Figure 5C). Similarly, NSC281245 binding resulted in reduced RMSF values (NSC281245 median RMSF ≈ 0.07 nm) with increased local residue flexibility compared to NSC36398 at residues around 470–487. The Rg analysis (see Supplementary File S1, Figure S6B) indicates that NSC281245 binding induces substantial compaction of the chain B RBD, whereas NSC36398 binding induces intermediate global compaction of the chain B RBD.

**Figure 6:**
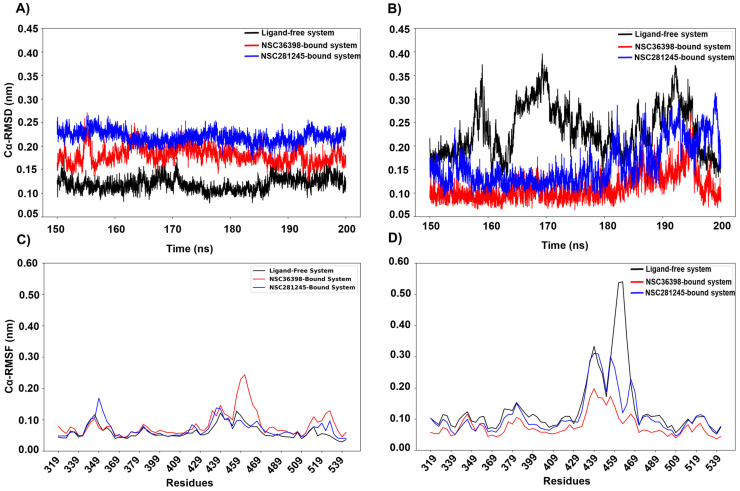
Captured Cα-RMSD plots for (**A**) chain A receptor-binding motif (residues 437–508), (**B**) chain B receptor-binding motif (residues 437–508), and Cα-RMSF plot (**C**) for chain A receptor-binding motif, (**D**) chain B receptor-binding motif.

Analyses of chain A and chain B RBMs revealed dynamic behaviour that broadly mirrors that of the larger RBD. Figure 6 shows the RBM dynamics for chains A and B in the HSPA8-spike protein complex. For chain A RBM, the ligand-free system displayed a relatively stable Cα-RMSD trace (Figure 6A; median RMSD ≈ 0.12 nm), whereas binding of NSC36398 (Figure 6A; median RMSD ≈ 0.19 nm) and NSC281245 (Figure 6A; median RMSD ≈ 0.22 nm) significantly increased the alpha-carbon structural deviations. Per-residue fluctuation analysis (Figure 6C) displayed broadly similar median RMSF values (median ≈ 0.06 nm) in both ligand-free and ligand-bound systems. The NSC36398-bound system RMSF plot displayed localised fluctuations with peaks at residues 473–486 and 495–505 (see Supplementary File S1, Figure S5B). By contrast, chain B RBM displayed an opposite dynamic behaviour to that of chain A. The ligand-free system exhibited the largest structural variability (median Cα-RMSD ≈ 0.17 nm; Figure 6B) and the most notable residue flexibility (median Cα-RMSF ≈ 0.08 nm; Figure 6D; see Supplementary File S1, Figure S5B), with dominant peaks around residues 473–486, compared to both the ligand-bound systems. NSC36398 binding reduced and reduced both the chain B RBM structural variability (median Cα-RMSD ≈ 0.11 nm; Figure 6B) and per-residue fluctuations (median Cα-RMSF ≈ 0.06 nm; Figure 6D; see Supplementary File S1, Figure S5B), suggesting increased stabilisation of the RBM. On the other hand, NSC281245 produced intermediate stabilisation of the RBM (median Cα-RMSD 0.16 nm; median Cα-RMSF ≈ 0.07 nm; Figure 6B,D).

These RBM observations are consistent with the observed RBD results. Chain B’s RBD and RBM displays larger ligand-free deviations that are stabilised by ligand binding, whereas chain A’s RBD is comparatively quiescent. Still, its RBM is susceptible to modest, ligand-induced local flexibility. This suggests that ligand binding results in modest local residue flexibility of chain A RBM, without altering the global flexibility of chain A RBD. Because the RBM encompasses the receptor-contacting surface of the RBD, the ligand-dependent changes observed in the HSPA8-spike protein complex could plausibly modulate receptor engagement and antigenic features, even if ACE2 is not present in the simulated complex [44].

### 2.4. NSC36398 and NSC281245’s Effect on the HSPA8-Spike Protein Complex Interaction

**Figure 7:**
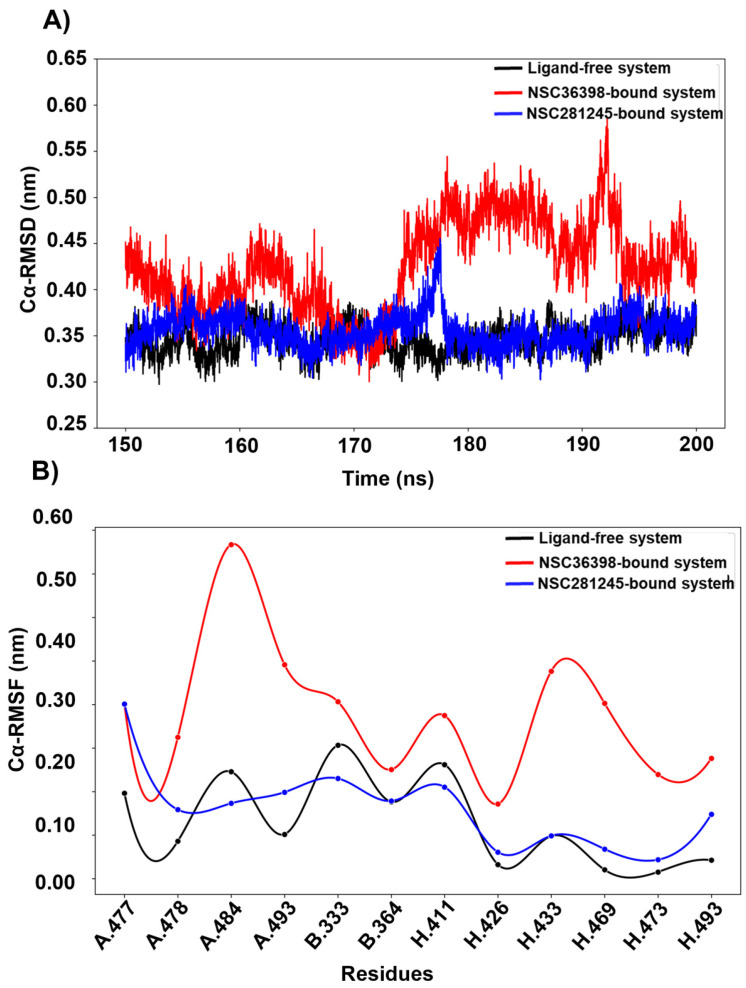
Captured (**A**) Cα-RMSD plot and, (**B**) Cα-RMSF plot for HSPA8-spike protein interaction interface.

The residues that define the HSPA8-spike protein interaction interface were isolated to understand the local structural dynamics. From molecular docking studies, the viral spike protein-associated residues at the interaction surface were identified as 478, 477, 484 and 493 from chain A, and 333 and 364 from chain B [19]. Those from the host HSPA8 were 411, 426, 433, 469, 473, and 493 [19]. Because of the direct involvement of the aforementioned HSPA8 residues at the interaction interface with the viral spike residues, we analysed per-residue Cα-RMSD (Figure 7A) and Cα-RMSF (Figure 7B) for these interface residues in ligand-free and ligand-bound systems.

The Cα-RMSD of the selected interface residues displayed increased significant structural deviations in the NSC36398-bound system (median RMSD ≈ 0.41 nm; see Supplementary File S1, Table S1) relative to the ligand-free (median RMSD ≈ 0.34 nm) and the NSC281245-bound systems (median RMSD ≈ 0.36 nm), as shown in Figure 7A. Per-residue mobility (Cα-RMSF; Figure 7B) shows increased residue flexibility in the NSC36398-bound system (median RMSF ≈ 0.12 nm), with the RMSF profile indicating residue fluctuations at residues that map to the RBM (spike chain A residues 478, 484 and 493), core RBD (spike chain B residues 333 and 364), the HSPA8 SBD β-domain and adjacent loops that form core elements of the peptide-binding groove [35,36,37,38,39,40,41]. NSC281245 (median RMSF ≈ 0.08 nm) produced a modest change to interface residue fluctuations from the ligand-free system baseline (median RMSF ≈ 0.07 nm). Taken together, the analysis indicates that NSC36398 perturbs the structural behaviour of interface residues by increasing both structural deviations and local residue flexibility at the contact patch between the HSPA8 and the SARS-CoV-2 spike protein potentially altering the stability of specific inter-protein contacts. By contrast, NSC281245 preserves the interface dynamic behaviour closely mimicking the ligand-free system dynamic behaviour.

### 2.5. NSC36398 and NSC281245 Binding Pocket Dynamics and Interactions

**Figure 8:**
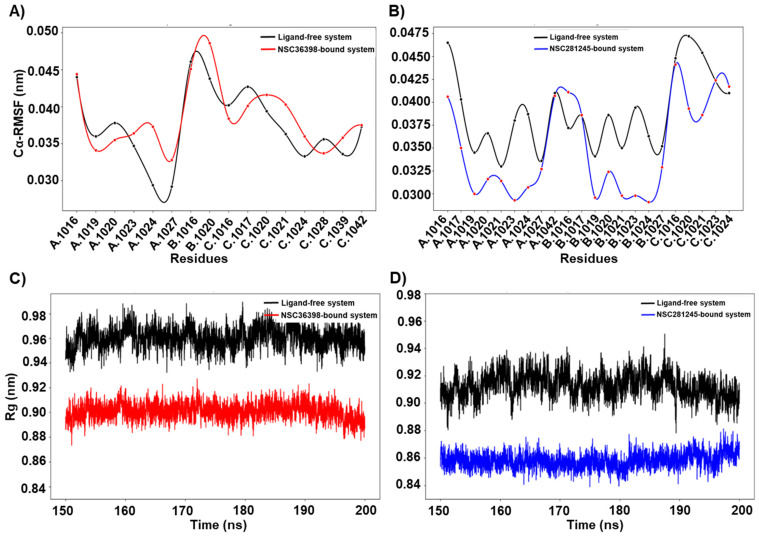
(**A**) Cα-RMSF line plot representation of the binding-pocket residues of the ligand-free system and NSC36398-bound system relative to their initial structures. (**B**) Cα-RMSF line plot representation of the binding-pocket residues of the ligand-free system and NSC281245-bound system relative to their initial structures. (**C**) Rg line plot representation of the ligand-free system and NSC36398 binding-pockets (**D**), and the ligand-free system and NSC281245 binding-pockets. Binding-pocket residues were defined as residues of any atom within 5 Å of the ligand in the initial frame structure.

Respective binding pockets were defined as residues within 5 Å of each ligand (Figure 8B,C) to account for major non-covalent interactions, such as hydrogen bonds and van der Waals interactions. Analysis of the binding pockets identified residues constituting part of the S2 subunit’s central helix (residues 986–1035) and the connector domain (residues 1036–1068) [19,44]. Both ligands were found to contact conserved residues across the spike protein chains, notably, ALA1016, ARG1019, ASN1023, LEU1024, and THR1027 from chain A and ALA1016, ALA1020, and LEU1024 from chain B. These shared residues are crucial for binding to HR1, suggesting that the ligands may interfere with the helix bundle formation required for viral entry [45,46].

Interestingly, amino acid residues ARG1019, ALA1020, and ASN1023 from chain A and GLU1017, LYS1028, ARG1039 (positively charged side chains), and PHE1042 from chain C were found to be in the vicinity of NSC36398’s binding pocket (see Figure 8B). NSC281245 had the following amino acid residues in its binding pocket: GLU1017, SER1021, PHE1042 from chain A, GLU1017, ALA1020, SER1021 from chain B; and SER1021, ASN1023 from chain C (see Figure 8C) [19,47]. GLU1017 (from chain A/B) and PHE1042 (chain A) interact with the ligand NSC281245. On the other hand, the ligand NSC36398 targets LYS1028 and ARG1039 from chain C, suggesting a strong specificity for amino acid residues and reliance on electrostatic interactions with positively charged residues [45,46,48].

With respect to the ligand-free system, the NSC36398-bound system, and the NSC281245-bound system, the binding pockets exhibited similar median Cα-RMSD trends (ligand-free ≈ 0.06 nm, ligand-bound ≈ 0.05 nm; see Supplementary File S1, Figure S7A), suggesting a stable binding site with subtle compaction, as expected of the S2 subunit of the spike protein [19,20,21,22,23,24,25,26,27,28,29,30,31,32,33,34,35,36,37,38,39,40,41,42,43,44,45,46,47,48,49]. Similarly, when assessing the Cα-RMSF for the NSC281245-bound system and the NSC36398-bound system (Figure 8A,B), only minor changes in residue fluctuation were observed, with those of the NSC281245 indicating a subtle decrease in residue flexibility relative to the apo system, suggesting a more stable binding, which agrees with the computed binding affinity results. We also observed that the NSC36398-bound system exhibited a slightly lower Rg value (median Rg ≈ 0.94 nm) than its ligand-free system (median Rg ≈0.95 nm; see Supplementary File S1, Figure S7B, indicating increased compaction due to favourable ligand binding. The NSC281245-bound system also demonstrated a relatively lower Rg value (median Rg ≈ 0.86 nm) than the ligand-free system (mean Rg ≈ 0.91 nm; see Figure 8D). The significant increase in compactness of the NSC281245 binding pocket relative to that of NSC36398 is potentially attributed to the higher number of hydrophobic amino acid residues constituting the binding pocket, which could facilitate tighter packing [50]. This clearly translates to a more efficient binding due to its hydrophobic nature. Regardless, rigidifying the binding pocket due to ligand binding may potentially dampen the helical breathing motions essential for prefusion to post-fusion transitions, thereby inhibiting viral membrane fusion [51,52].

### 2.6. Insights into NSC36398 and NSC281245 Hydrogen Bonds with Their Respective Binding Sites

**Figure 9:**
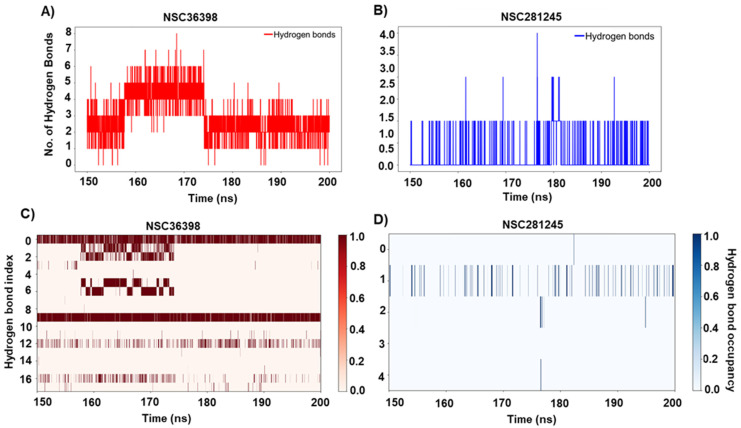
Number of hydrogen bonds detected between (**A**) HSPA8-spike protein complex and NSC36398, and (**B**) HSPA8-spike protein complex and NSC281245, over the 150–200 ns trajectory. Hydrogen-bond occupancies detected between the HSPA8-spike protein complex and the (**C**) NSC36398 and (**D**) NSC281245 ligands, respectively. The associated hydrogen bond donors and acceptors are indicated in Supplementary File S1, Table S2 (NSC36398) and Table S3 (NSC281245), as indexed.

According to Gowo (2021) [53] and Spassov (2024) [54], hydrogen bonds are among the main molecular structures stabilising intermolecular interactions. Figure 9A,C demonstrated a dynamic change in hydrogen bond interactions between NSC36398 and the binding pocket. The presence of these hydrogen bonds throughout the converged portion of the simulation underscores the importance of these interactions in anchoring the NSC36398 ligand within the binding pocket (Figure 9A). The high-occupancy hydrogen bonds (Figure 9C), particularly SER1021:OG-LG31145:O3 and LG31145:O1-ASN1023:ND2 (see Supplementary File S1, Table S2), likely contribute the most to the stability of the complex [53]. The transient hydrogen bond interactions of NSC36398 and the binding site (S2 subunit) potentially facilitate induced-fit adaptations, as explicitly discussed by Csermely et al. (2010) [55], allowing the ligand to induce allosteric-like effects through local perturbations that subtly propagate to distal domains within the protein complex, a hypothesis that aligns with the observed results from RMSF.

In contrast to NSC36398 (which averaged 3–4 persistent bonds), NSC281245 formed fewer and more transient hydrogen bonds (Figure 9B,D), which suggests a weaker network of polar interactions. In this case, ARG1019NH2-LG41145O1 exhibited the highest consistency, as indicated by the hydrogen bond (index 1, see Supplementary File S1, Table S3). This observation is consistent with the ligand’s docking affinity towards the HSPA8-spike protein observed in our previous study [19], which is not unexpected given its hydrophobic properties and size. The observed spikes in H-bond count at ~175–180 ns indicate momentary adjustments, in which the ligand briefly shifted to maximise polar contacts [56,57].

### 2.7. Implications of Binding Free Energy Estimates for the HSPA8-Spike Protein Complexes in the Presence and Absence of NSC36398 and NSC281245

Binding free energy calculations yielded quantitative insights into two key interactions: firstly, ligand affinities within the HSPA8-spike-ligand ternary complex, and ligand-induced modulation of the HSPA8-spike protein–protein interface via gmx_MMPBSA. Both approaches converged on strongly favourable binding energetics (ΔG < 0) (see Supplementary File S1, Table S4) [47,48], revealing an interesting dual mechanism underlying small-molecule allosteric-like inhibition.

**Figure 10:**
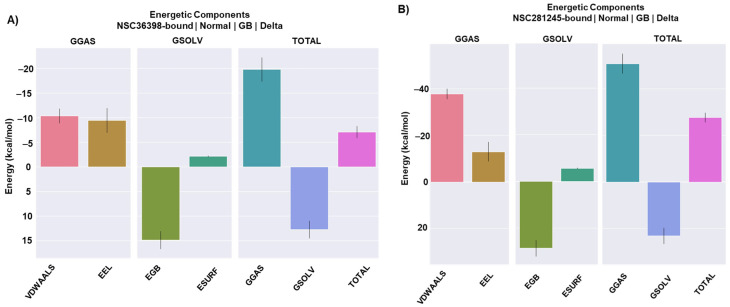
MM/GBSA energetic decomposition of each ligand in their respective S2 subunit binding pocket in (**A**) NSC36398-bound and (**B**) NSC281245-bound systems calculated using gmx_MMPBSA.

NSC36398 binds modestly stable to the S2 binding site (ΔG_bind_ = −7.07 ± 0.04 kcal/mol) through persistent non-covalent bonds (Figure 10A). This negative value confirms the stability of NSC36398 within the protein complex, consistent with the fundamental principle that a negative ΔG_bind_ indicates spontaneous binding and complex stability [58]. Energy decomposition of the free energy revealed that the ΔG_bind_ of NSC36298 at the binding pocket was driven by strong van der Waals (Δ_VDWAALS_ = −10.35 kcal/mol) and electrostatic charged/polar (Δ_EEL_ = −9.45 kcal/mol) interactions (Figure 10A). These, combined with gas-phase interactions (Δ_GGAS_ = −19.80 kcal/mol), greatly stabilise the complex. However, the favourable gas-phase interactions were neutralised by high polar solvation penalties. The polar solvation energy (Δ_EGB_) showed an unfavourable contribution of 14.88 kcal/mol, indicating the energetic cost associated with water removal from polar groups participating in binding interactions [58]. Conversely, the nonpolar solvation energy (Δ_ESURF_) component contributed a modest favourable energy of −2.15 kcal/mol (Figure 10A). This, in turn, indicates limited burial of hydrophobic surfaces. The net solvation free energy (ΔG_solv_ = Δ_EGB_ + Δ_ESURF_) was unfavourable at 12.72 kcal/mol, demonstrating how solvation costs counteract gas-phase stabilisation. The large ΔEGB value limited the thermodynamic stability of NSC36398, suggesting that NSC36398 consists of a few hydrophobic contacts [59,60]. This is consistent with the conducted hydrogen bond analysis, which displayed 3–4 persistent hydrogen bonds that anchor NSC36398 (see Figure 9A,C). This binding notably destabilises the native HSPA8-spike protein interface (see Figure 7).

The ligand-free HSPA8-spike complex displayed a robust affinity (ΔG_bind_ = −67.44 ± 0.46 kcal/mol; see Supplementary File S1, Table S5). These findings align with and provide quantitative support for the domain-level binding of inducible HSP70 SBD to the SARS-CoV-2 spike RBD previously reported by Joshi et al. (2024) [8]. However, the interaction interface’s stability dropped substantially upon NSC36398 binding (ΔG_bind_ = −34.39 ± 0.54 kcal/mol; see Supplementary File S1, Table S5). Critically, the destabilisation of the HSPA8-spike protein interface in the NSC36398-bound system directly correlated with the observed reduced protein binding affinity. This suggests that small-molecule NSC36398 may be an allosteric-like inhibitor, disrupting the distal functional interface (HSPA8-spike protein) through long-range dynamic effects originating from its S2 binding site. This mechanism of allosteric-like disruption and interference aligns with the established models of allosteric communication in the spike protein [61].

In contrast, NSC281245 strongly binds to the S2 binding site (ΔG_bind_ = −27.63 ± 0.07 kcal/mol). NSC281245 engaged a more extensive interface on the S2 binding pocket surface due to its size, maximising hydrophobic contacts. The energy decomposition revealed that the ΔG_bind_ of NSC281245 at the S2 binding pocket was primarily driven by strong van der Waals (ΔV_DWAALS_ = −37.80 kcal/mol) and electrostatic charged/polar (Δ_EEL_ = −12.99 kcal/mol) interactions (see Supplementary File S1, Table S4; Figure 10B). Hence, the large negative van der Waals contribution indicated that hydrophobic contacts dominated NSC281245’s binding to the S2 binding pocket [60,62]. Additionally, with the gas-phase interactions (Δ_GGAS_ = −50.79 kcal/mol) of NSC281245, the HSPA8-spike-NSC281245 complex was notably stabilised. NSC281245 displayed a Δ_EGB_ of 28.72 kcal/mol, which partially counteracts the previously mentioned van der Waals and electrostatic interactions. It also exhibited a Δ_ESURF_ of −5.56 kcal/mol, which adds a small favourable component to the binding free energy (see Supplementary File S1, Table S4; Figure 10B). Overall, this finding aligns with the hydrogen bond analysis performed, which revealed only one persistent hydrogen bond (Figure 6D), suggesting that hydrophobic interactions are central to the complex’s stability.

Converse to NSC36398’s binding effects on the protein–protein affinity of the HSPA8-spike complex, NSC281245 binding appears to preserve the near-native interface (ΔG_bind_ = −62.26 ± 0.56 kcal/mol; see Supplementary File S1, Table S5), which suggests that NSC281245 may induce minimal-to-no disruptive effects. Therefore, these findings highlight NSC36398 as a promising allosteric-like inhibitor for the HSPA8-spike protein complex.

## 3. Materials and Methods

This study performed ligand-free and ligand-bound MD simulations using GROMACS v2018.6 [63] with structures prepared from previous molecular docking experiments [19]. Ligand-free MD simulations comprised the predicted protein complex (HSPA8-spike protein complex) without a ligand generated using BioLuminate v4.6, with the best docked pose selected based on PIPER cluster size, PIPER pose energy and PIPER pose score. The ligand-bound MD simulations included a single ligand, either NSC36398-bound or NSC281245-bound, generated from molecular docking simulations using Maestro v13.1. The potential binding pocket on the docked protein complex was identified using the SiteMap tool within Maestro v13.1 and was used to generate a grid box which was in turn used in performing targeted protein complex-ligand docking. Full protocol details for the protein–protein and protein–ligand docking studies including protein and ligand preparation, forcefield, parameters used and selection criteria used in determining the best docked poses are found in our publication, Navhaya et al. (2024) [19]. Following established practice for interface-centric studies [25,26,27], we used the pre-formed HSPA8-spike protein complex and applied identical minimisation/equilibration protocols across apo and ligand-bound systems to isolate ligand-specific perturbations.

### 3.1. MD System Preparation: Ligand-Free and Ligand-Bound System Parameter Definition

The AMBER99SB-ILDN force field [64] was assigned to the systems within GROMACS using the *gmx pdb2gmx* command [63]. ACPYPE v.2023.10.27 was used to produce GROMACS-compatible parameter and topology files for the ligands [65,66]. Protein–ligand *.gro* files were built by manually merging the ligand atoms into the prepared protein *.gro* file, and the system topology file was updated to integrate ligand parameters. The ligand-free and ligand-bound systems were placed in a triclinic box with a minimum solute-box distance of 1.8 nm, solvated with TIP3P water. Systems were also neutralised and adjusted to 0.15 M NaCl using *gmx genion* [63,67]. Energy minimisation of both systems was performed using the *gmx mdrun* command. The systems were relaxed using the steepest descent algorithm, with a force tolerance of 1000 kJ/mol/nm and an upper limit of 50,000 steps (cutoff-scheme = Verlet; rcoulomb = 1.0; rvdw = 1.0). The systems were equilibrated and subsequently further run on the Lengau Centre for High-Performance Computing (CHPC) cluster. Using the NVT ensemble, temperature equilibration in the systems was performed for 100 ps with V-rescale coupling at 300 K (tau_t = 0.1 ps). For each system we performed three independent replicates; initial velocities were generated from a Maxwell distribution at 300 K (gen_vel = yes; gen_seed = −1). The systems were then pressure equilibrated using the NPT ensemble by applying the Berendsen barostat for 100 ps at 1 bar (tau_p = 2.0 ps, ref_p = 1.0 bar, compressibility = 4.5 × 10^−5^ bar^−1^), with a maximum of 50,000 steps [48]. The LINCS method was used to restrain the bond lengths. Long-range electrostatics were handled using the Particle Mesh Ewald (PME) method, with short-range nonbonded interactions treated with cut-off values of 1.0 nm for the ligand-bound and ligand-free systems, respectively [68,69,70]. All-atom MD productions were carried out in triplicate using the *gmx mdrun* command for 200 ns with a timestep of 2 femtoseconds (fs). Parrinello-Rahman barostat was used (tau_p = 2.0 ps, ref_p = 1.0 bar, compressibility = 4.5 × 10^−5^ bar^−1^) [71]. The exact *.mdp* parameter files used for energy minimisation, equilibration (NVT and NPT) and MD production are provided in Supplementary File S2A–G.

### 3.2. Post-MD Analysis

After the 200 ns simulations were completed, trajectories were post-processed with *gmx trjconv*, where the periodic boundary conditions were removed, and post-MD simulation analyses were conducted. The last 50 ns (150 ns to 200 ns) of the three systems, namely, the ligand-free, the NSC36398-bound, and the NSC281245-bound systems, were used in this analysis to demonstrate the equilibration phase and reproducibility of the results.

#### 3.2.1. Structural Analysis

Structural analysis of the ligand-free system, NSC36398-bound system, and NSC281245-bound system was conducted using GROMACS tools (*gmx rms*, *gmx rmsf*, and *gmx gyrate*) [58]. Convergence was assessed by observing RMSD and calculating block averaging for the selected ligand-free RMSD time series using an in-house ad hoc script (see Supplementary File S2H). For block sizes 1 to n_frames, the script computes block averages and the SEM of the block averages (SEM=s_block/√N_blocks). The script outputs are provided in the Supplementary Data (see Supplementary File S1). All plots for structural metrics (RMSD, RMSF, and Rg) were generated using matplotlib and seaborn. RMSD, RMSF, and Rg were calculated to evaluate and assess the conformational changes, stability, flexibility, and compactness of the proteins and their components [72,73]. Because RMSD, RMSF and Rg time-series deviated from normality, central tendency is reported as median (see Supplementary File S2I). Functional domain characterisation of the HSPA8 SBD, the SARS-CoV-2 spike RBD comprising chain A and chain B RBD, as well as the binding sites of the ligand of interest (i.e., residues within 5 Å of either ligand), was performed on the last 50 ns of the converged phase of the trajectories.

#### 3.2.2. Hydrogen Bond Calculation Protocol

The hydrogen bond calculation was performed to analyse the interactions between the protein complex S2 subunit binding site and each of the ligands, NSC36398 and NSC281285. The calculation was conducted using the *gmx hbond* command [63], with a cut-off distance of 0.35 nm and an angle of 30°, using the converged data.

#### 3.2.3. Binding Free Energy Calculations

##### Protein–Ligand Binding Free Energy Calculations

Binding free energy calculations of the ligand-bound systems were performed using the MM/GBSA approach within the gmx_MMPBSA pipeline (v1.6.3) [74,75]. This method calculated the binding free energy (ΔG_bind_) as the difference between the free energy of the complex (G_complex_) and the sum of free energies for the isolated receptor (G_receptor_) and ligand components (G_ligand_) as shown in the equation below:

(1) $$∆G_{bind}=G_{complex}-\left(G_{receptor}+G_{ligand}\right).$$

Each G included Molecular Mechanics (MM), polar solvation (GB), and nonpolar solvation (SA). The analysis used 1001 frames extracted from 190 to 200 ns of the simulation trajectory. Topologies generated in GROMACS (AMBER99SB for protein; GAFF for ligand) were converted to AMBER compatibility, and solvation effects were modelled using the Generalised Born model (igb = 5, mbondi2 radii) alongside a SASA-based nonpolar term (γ = 0.0072 kcal/molÅ^−2^). A single-trajectory protocol ensured identical conformational sampling of the complex, receptor, and ligand states, minimising statistical noise. Per-frame energy components (MM, G_GB, G_SA) were computed in parallel (8 CPUs), while entropy contributions were omitted, resulting in a purely enthalpic ΔG_bind_ [62,63,64,65,66,67,68,69,70,71,72,73,74,75,76].

##### Protein–Protein Binding Free Energy Calculations

Binding free energies (ΔG_bind_), defining the interaction between HSPA8 SBD (residues 349–509) and the SARS-CoV-2 spike protein RBD (319–541) from chains A and B, were estimated using the MM/GBSA approach within the gmx_MMPBSA pipeline (v1.6.3) [45]. The calculations employed the Generalised Born model, with an internal dielectric constant of 1.0 and an external dielectric constant of 78.5, over the last 10 ns of the simulation. The binding free energy (ΔG_bind_) was calculated as the difference between the free energy of the complex (G_complex_; SBD-RBD complex) and the sum of free energies for the isolated receptor (G_receptor_; SBD residues) and ligand components (G_ligand_; RBD residues from chain A and B) as shown by equation in Section Protein–Protein Binding Free Energy Calculations [62,63,64,65,66,67,68,69,70,71,72,73,74,75,76].

## 4. Conclusions

The persistent threat of COVID-19 highlights the urgent need for innovative and effective therapeutic strategies. This study focused on the HSPA8-spike protein complex as a potential target, employing in silico techniques to screen, identify, and repurpose small-molecule compounds for the development of COVID-19 drugs. MD simulations were used to validate previous docking experiments that identified small-molecule compounds with stable, viral-preferential binding and high binding affinities. The protein–ligand docking experiments had identified small molecules NSC36398 and NSC281245 as potential compounds exhibiting stable interactions and the best binding affinities towards key regions within the spike protein’s conserved S2 subunit, particularly in complex with HSPA8. In the current study, we confirmed the formation of stable complexes through MD simulations and revealed distinct ligand effects on the complex. NSC36398 induced significant, thermodynamically favourable allosteric modulations, affecting the flexibility, stability, and interaction surface of HSPA8 and the viral domains. Furthermore, the drug-like properties provide a strong rationale for further experimental validation. This work has therefore identified NSC36398 as a promising repurposed candidate targeting the HSPA8-spike protein complex, indicating the potential of the hit compound to serve as a quantitative framework for rational design of drug compounds that stably engage key S2 subunit residues and effectively destabilise this HSPA8-spike interaction.

While our interface-focused study isolates ligand-dependent perturbations, it lacks systematic negative controls (isolated protein runs and non-binding ligands) to confirm binding specificity, positive controls (benchmarking against known HSPA8/spike inhibitors), and experimental validation of the results obtained in this study. Future work will likely employ in vitro inhibitor screening assays to quantify and validate the impact of the small compound on HSPA8-spike binding. Additionally, experimental benchmarking against known HSPA8/spike inhibitors will be performed to assess the relative potency and selectivity of the identified compound. Afterwards, pseudo-virus entry inhibitor assays in relevant cell models should be conducted to confirm functional blockade. Lastly, rational structure-based optimisation of the identified NSC36398 compound scaffold should be undertaken to enhance drug potency and selectivity.

## Figures and Tables

**Table 1:** Two-dimensional representations and key structural insights of the small-molecule compounds of interest.

Compound ID	Structure	Key Structural Insight
NSC36398	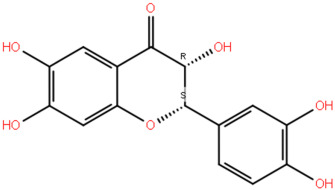	Compact, rigid polyphenolic scaffold potentially forming a stable π-π stacking and directional hydrogen bonding. Potentially more reliable for establishing several stabilising interactions.
NSC281245	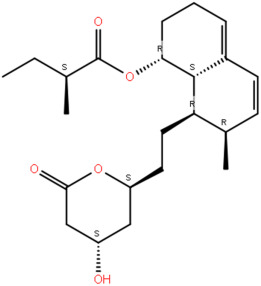	Hydrophobic polycyclic core plus polar groups. Potentially favours van der Waals or hydrophobic interactions. It likely has low mobility in the hydrophobic pocket.

## Data Availability

The original contributions presented in this study are included in the article and Supplementary Materials. Further inquiries can be directed to the corresponding author.

## Supplementary Materials

The following supporting information can be downloaded at: https://www.mdpi.com/article/10.3390/ijms27104288/s1.

## Abbreviations

The following abbreviations are used in this manuscript:

ACE2	Angiotensin-Converting Enzyme 2
ACPYPE	AnteChamber Python Parser Interface
ADMET	Absorption, Distribution, Metabolism, Excretion and Toxicity
AMBER	Assisted Model Building with Energy Refinement
CHPC	Centre of High Computing
COVID-19	Coronavirus Disease 2019
GROMACS	GROningen MAchine for Chemical Simulations
HSP	Heat shock protein
HSPA8	Heat Shock Protein Family A Member 8
IBV	Infectious Bronchitis Virus
JEV	Japanese Encephalitis Virus
MD	Molecular Dynamics
MM/GBSA	Molecular Mechanics Generalised Born Surface Area
MM-PBSA	Molecular Mechanics Poisson-Boltzmann Surface Area
RBD	Receptor-Binding Domain
RBM	Receptor-Binding Motif
Rg	Radius of Gyration
RMSD	Root Mean Square Deviation
RMSF	Root Mean Square Fluctuation
SARS-CoV-2	Severe Acute Respiratory Syndrome Coronavirus 2
SARS-CoV-2 Mpro	Severe Acute Respiratory Syndrome Coronavirus 2 Membrane Protease
SBD	Substrate-Binding Domain
