# Supplementary material for: In-Depth Molecular Dynamics Simulations Reveal Ligand-Induced Modulations of the HSPA8-SARS-CoV-2 Spike Protein Interaction
Source: Int J Mol Sci. 2026 May 12;27(10):4288. doi: 10.3390/ijms27104288 (PMC13207655; doi:10.3390/ijms27104288)
Supplement: Supplementary file 1 [file ijms-27-04288-s001.zip › ijms-3866908-Supplementary_File_S1.pdf]

Supplementary Materials for

In-Depth Molecular Dynamics Simulations Reveal Ligand-Induced Modulations of the HSPA8-SARS-CoV-2 Spike Protein Interaction

Liberty T. Navhaya [1], Mokgerwa Z. Monama [1], Thabe M. Matsebatlela [1] and Xolani H. Makhoba [2,*]

[1] Department of Biochemistry, Microbiology, and Biotechnology, University of Limpopo, Turfloop Campus, Sovenga 7270, South Africa; 202417284@myturf.ul.ac.za (L.T.N.); mokgerwa.monama@ul.ac.za (M.Z.M.); thabe.matsebatlela@ul.ac.za (T.M.M.)

[2] Department of Life and Consumer Sciences, College of Agriculture and Environmental Sciences, University of South Africa (UNISA), Florida Campus, Roodepoort 1709, South Africa

* Correspondence: makhoxh@unisa.ac.za

Note: The material contained herein is supplementary to the article named in the title and published in the International Journal of Molecular Sciences

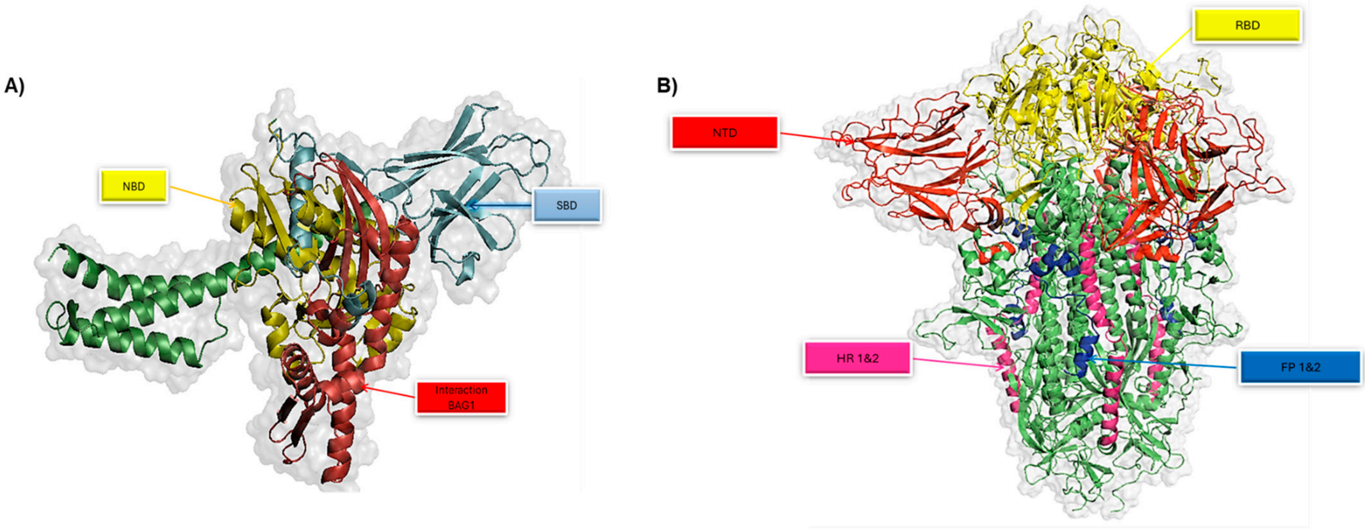

Figure S1: Three-dimensional structures of the **(A)** human HSPA8 indicating the N-terminal nucleotide-binding domain (NBD; residue positions 2-386) and Substrate-binding domain (SBD; residue positions 349-509), and the **(B)** SARS-CoV-2 spike protein the receptor-binding domain (RBD; residue positions 319-541), C-terminal domain (residue positions 334-527), N-terminal domain (NTD; residues 14-303), fusion peptides (FP1 and FP2), heptad repeat units (HR1 and HR2)Adapted from Navhaya et al. (2024)[19].

https://doi.org/10.3390/xxxxx

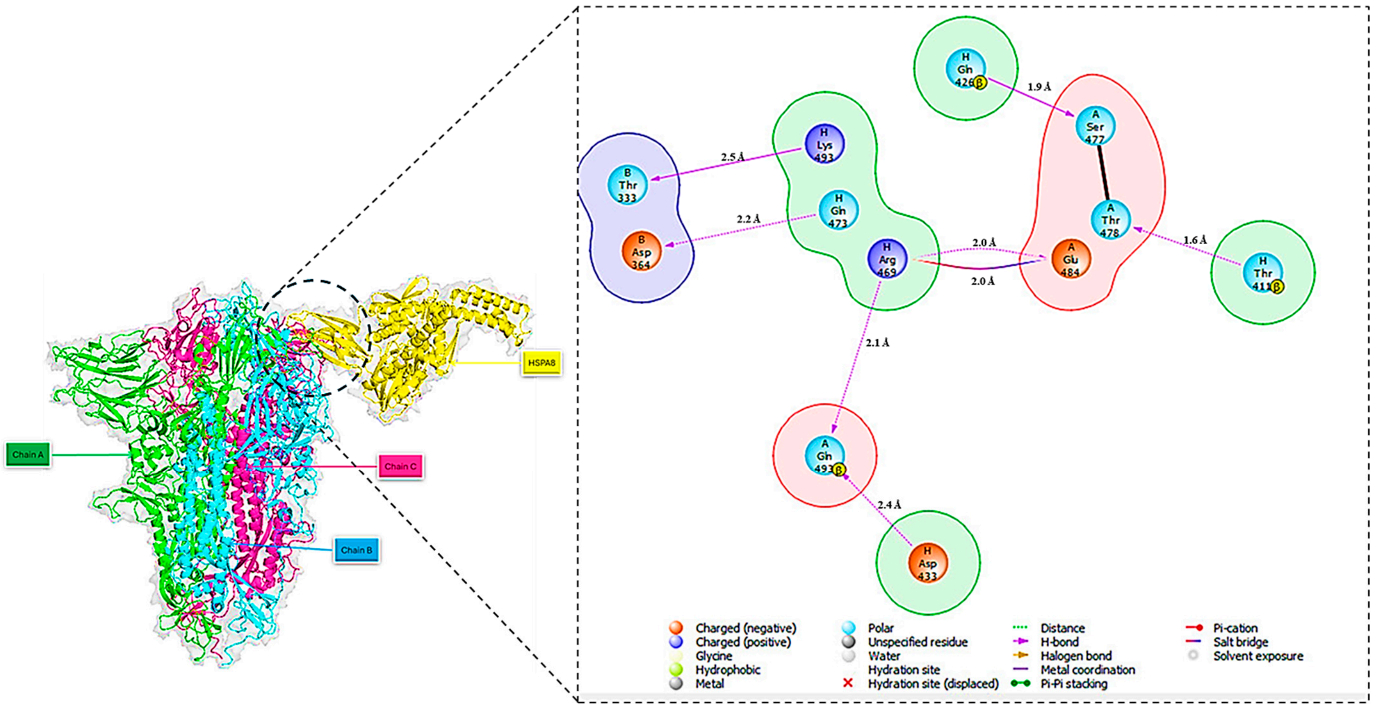

Figure S2: Interface residue interactions formed between the HSPA8 substrate-binding domain (SBD; residues 405-495) and the SARS-CoV-2 receptor-binding domain (RBD) following protein-protein docking. The HSPA8 SBD interacts with the RBD from chain A (residues 455-495) and chain B (residues 333-371) through multiple hydrogen bonds and a single salt bridge, with measured bond distances ranging from **1.6 to 2.5 Å**. Adapted from Navhaya et al. (2024)[19].

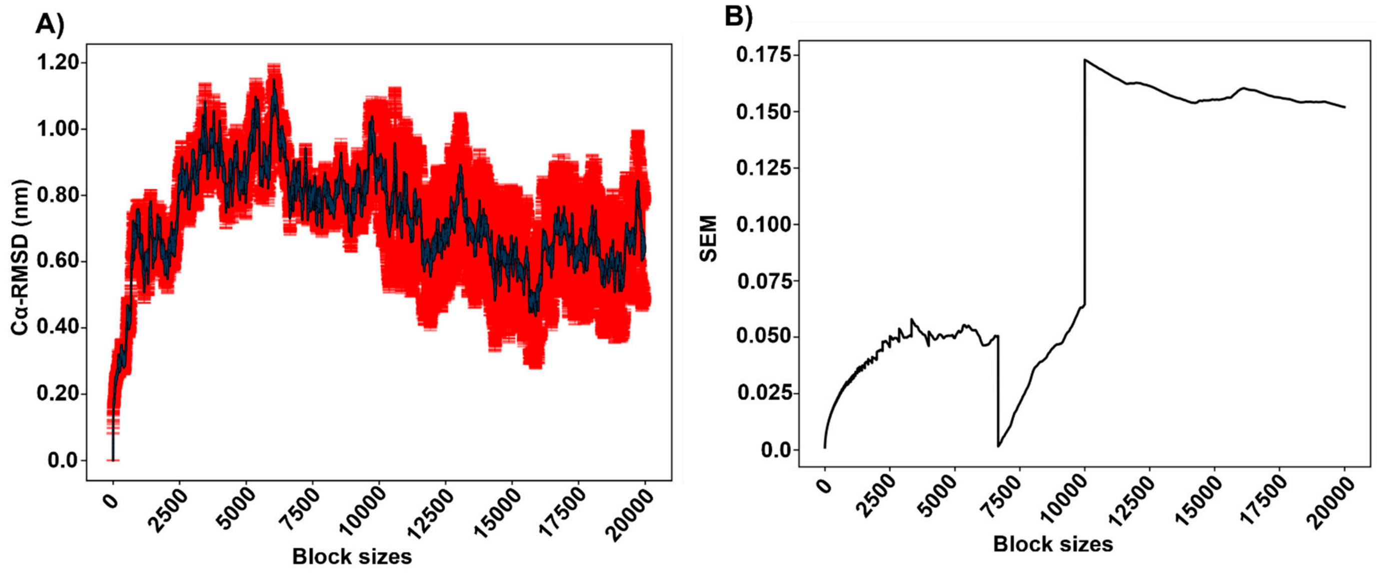

Figure S3: (A) Root mean square deviation (RMSD) plotted against block sizes, showing fluctuations over varying block sizes with a black line representing the structural deviations and the red shading indicating variability, **(B)** Standard error of the mean (SEM) plotted against block sizes, illustrating the change in SEM as block sizes increase.

Table S1: Consolidated statistical summary comparing ligand-free and ligand-bound Cα-RMSD, Cα-RMSF, and Rg data using the Mann-Whitney U test.

Protein structure + applied metric	Comparison	Median	IQR	p-value	Significance (p-value < 0.05 threshold)
**HSPA8-spike complex RMSD**	Ligand-free vs NSC36398-bound	0.636365<br>0.721867	0.564775-0.667102<br>0.702632-0.772053	0.000495534	Yes
	Ligand-free vs NSC281245-bound	0.636365<br>0.694539	0.564775-0.667102<br>0.672934-0.718281	0.0100445	Yes
**HSPA8-SBD RMSD**	Ligand-free vs NSC36398-bound	0.222720<br>0.264016	0.208106-0.236099<br>0.208106-0.236099	0.0021645	Yes
	Ligand-free vs NSC281245-bound	0.227134<br>0.220330	0.216562-0.235462<br>0.207532-0.240657	0.492464	No
**HSPA8-SBD RMSF**	Ligand-free vs NSC36398-bound	0.068900<br>0.081900	0.054950-0.082600<br>0.069900-0.101650	0.0240219	Yes
	Ligand-free vs NSC281245-bound	0.063800<br>0.069400	0.053900-0.081600<br>0.058100-0.089900	0.317836	No
**HSPA8-SBD Rg**	Ligand-free vs NSC36398-bound	2.200490<br>2.175160	2.193515-2.205805<br>2.168485-2.182645	1.25e-06	Yes
	Ligand-free vs NSC281245-bound	2.199125<br>2.206560	2.194900-2.201935<br>2.198975-2.212718	0.035045	Yes
**Chain A RBD RMSD**	Ligand-free vs NSC36398-bound	0.192278<br>0.184385	0.190540-0.199388<br>0.181703-0.190657	0.0280651	Yes
	Ligand-free vs NSC281245-bound	0.192278<br>0.206663	0.190540-0.199388<br>0.202878-0.220336	0.000552853	Yes
**Chain A RBD RMSF**	Ligand-free vs NSC36398-bound	0.061000<br>0.062000	0.050150-0.074600<br>0.052350-0.085650	0.49947	No
	Ligand-free vs NSC281245-bound	0.060100<br>0.057800	0.044225-0.074200<br>0.050500-0.076175	0.738902	No
**Chain A RBD Rg**	Ligand-free vs NSC36398-bound	2.227570<br>2.203550	2.221750-2.229740<br>2.199980-2.211280	2.30756e-06	Yes
	Ligand-free vs NSC281245-bound	2.223445<br>2.225380	2.217763-2.227607<br>2.221850-2.229300	0.103913	No
**Chain B RBD RMSD**	Ligand-free vs NSC36398-bound	0.173253<br>0.113113	0.157117-0.193540<br>0.108346-0.120754	5.38677e-05	Yes
	Ligand-free vs NSC281245-bound	0.173253<br>0.160245	0.160471-0.204843<br>0.150037-0.161875	0.309524	No
**Chain B RBD RMSF**	Ligand-free vs NSC36398-bound	0.078250<br>0.063000	0.061425-0.092425<br>0.053275-0.086550	0.142691	No
	Ligand-free vs NSC281245-bound	0.078250<br>0.070750	0.061425-0.092425<br>0.057500-0.096600	0.605849	No

**Chain B RBD Rg**	Ligand-free vs NSC36398-bound	2.206630 2.198465	2.199295-2.214215 2.194365-2.204363	8.57205e-05	Yes
	Ligand-free vs NSC281245-bound	2.210545 2.179540	2.199515-2.217280 2.177335-2.184690	1.49564e-06	Yes
**Chain A RBM RMSD**	Ligand-free vs NSC36398-bound	0.123631 0.178578	0.107731-0.129964 0.168634-0.196149	2.20382e-10	Yes
	Ligand-free vs NSC281245-bound	0.113434 0.223045	0.106394-0.120440 0.210033-0.232204	3.32734e-09	Yes
**Chain A RBM RMSF**	Ligand-free vs NSC36398-bound	0.061000 0.062000	0.050150-0.074600 0.052350-0.085650	0.49947	No
	Ligand-free vs NSC281245-bound	0.060100 0.057800	0.044225-0.074200 0.050500-0.076175	0.738902	No
**Chain A RBM Rg**	Ligand-free vs NSC36398-bound	1.554580 1.551310	1.548410-1.555810 1.548730-1.571070	0.73044	No
	Ligand-free vs NSC281245-bound	1.556545 1.540695	1.549275-1.562870 1.531935-1.549853	0.0206682	Yes
**Chain B RBM RMSD**	Ligand-free vs NSC36398-bound	0.221590 0.094807	0.176277-0.240897 0.094807	0.0001554	Yes
	Ligand-free vs NSC281245-bound	0.217079 0.167374	0.215959-0.267546 0.147659-0.193547	0.0952381	No
**Chain B RBM RMSF**	Ligand-free vs NSC36398-bound	0.106300 0.063700	0.100700-0.113000 0.058200-0.098700	0.00777458	Yes
	Ligand-free vs NSC281245-bound	0.110300 0.099100	0.102100-0.125825 0.095300-0.103475	0.104895	No
**Chain B RBM Rg**	Ligand-free vs NSC36398-bound	1.574595 1.537325	1.559583-1.588930 1.532198-1.552717	4.95534e-05	Yes
	Ligand-free vs NSC281245-bound	1.590370 1.540695	1.586785-1.600618 1.531935-1.549853	0.0001554	Yes
**Interaction Interface RMSD**	Ligand-free vs NSC36398-bound	0.343605 0.412419	0.339861-0.349637 0.382763-0.456131	0.00407925	Yes
	Ligand-free vs NSC281245-bound	0.342829 0.358306	0.333655-0.349594 0.352394-0.363505	0.000326003	Yes
**Interaction Interface RMSF**	Ligand-free vs NSC36398-bound	0.069850 0.120350	0.056325-0.082575 0.118875-0.124950	0.0285714	Yes
	Ligand-free vs NSC281245-bound	0.069850 0.080900	0.056325-0.082575 0.073175-0.091625	0.485714	No
**NSC36398 Binding Pocket RMSD**	Ligand-free vs NSC36398-bound	0.058186 0.064885	0.054578-0.063683 0.062578-0.071108	0.00147772	Yes

**NSC36398 Binding Pocket RMSF**	Ligand-free vs NSC36398-bound	0.058186 0.037850	0.054578-0.063683 0.035950-0.042300	0.720901	No
**NSC36398 Binding Pocket Rg**	Ligand-free vs NSC36398-bound	0.945176 0.938606	0.940350-0.949551 0.933979-0.942300	2.52143e-08	Yes
**NSC281245 Binding Pocket RMSD**	Ligand-free vs NSC281245-bound	0.058065 0.050493	0.050269-0.060522 0.045838-0.055545	0.022447	Yes
**NSC281245 Binding Pocket RMSF**	Ligand-free vs NSC281245-bound	0.038650 0.035850	0.037050-0.042375 0.031375-0.040725	0.328205	No
**NSC281245 Binding Pocket Rg**	Ligand-free vs NSC281245-bound	0.884418 0.875533	0.880259-0.888475 0.872382-0.878533	2.72867e-14	Yes

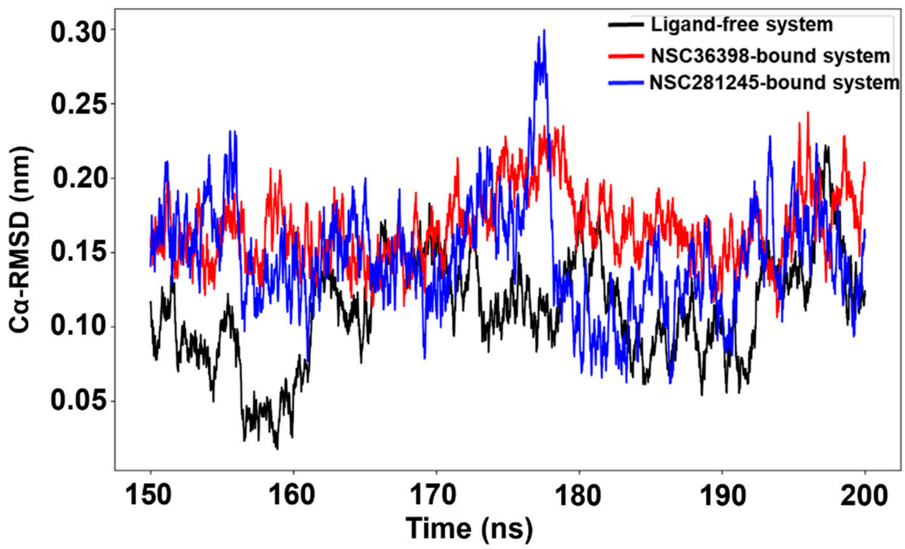

Figure S4: Estimated Cα-RMSD line plots of the ligand-free system, NSC36398-bound system, and NSC281245-bound system, generated using *gmx rms* for the last 50 ns of the converged trajectories.

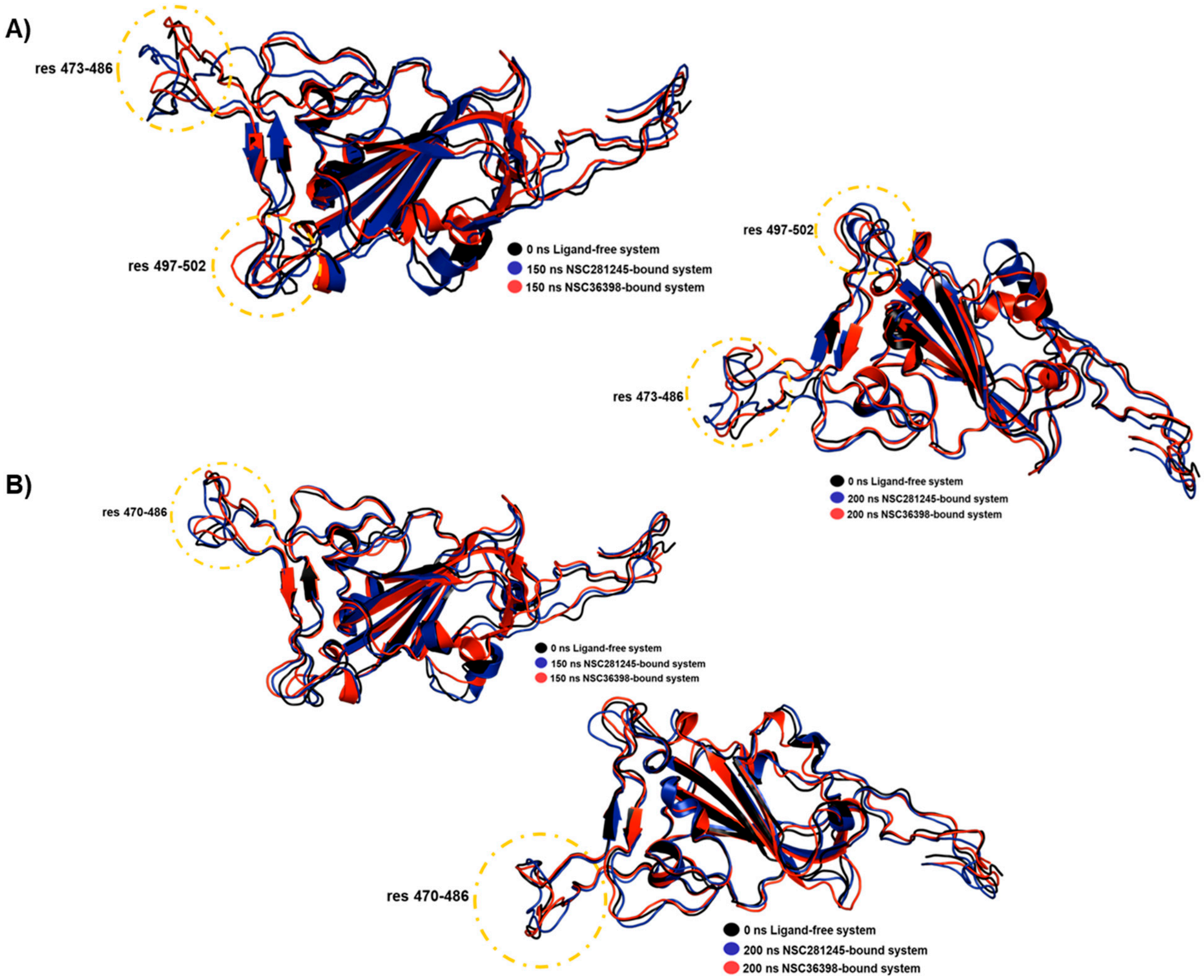

Figure S5: Structural comparison of the ligand-free reference (black; cartoon representation) at 0 ns, and the NSC36398-bound system (red; cartoon representation) and NSC281245-bound system (blue; cartoon representation) RBDs at 150 and 200 ns form **(A)** spike protein chain A RBD and **(B)** spike protein chain B RBD.

https://doi.org/10.3390/xxxxx

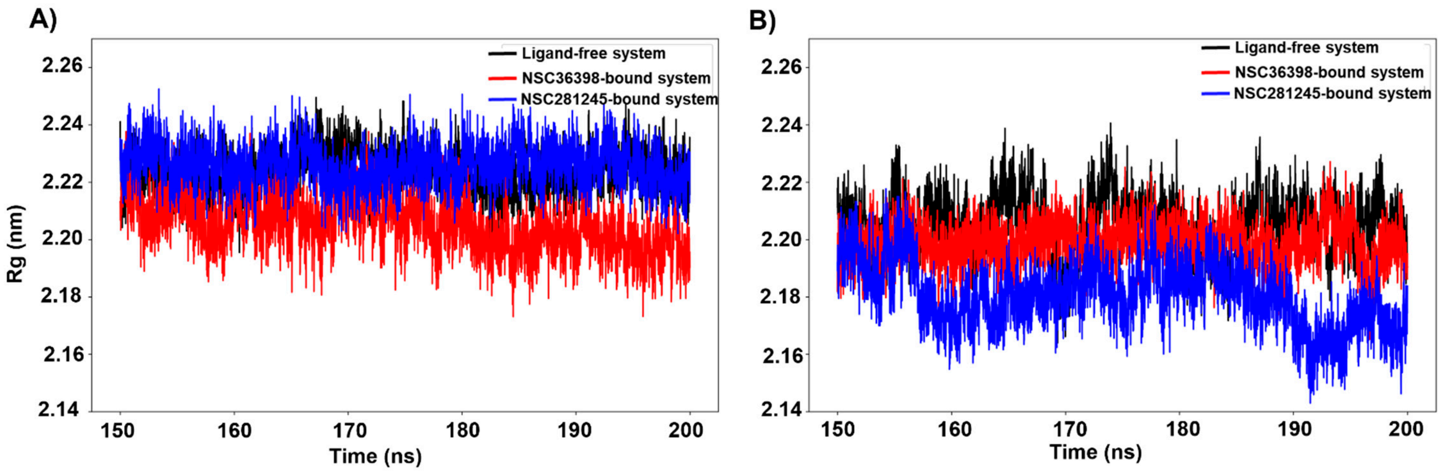

Figure S6: Captured radius of gyration of **(A)** SARS-CoV-2 chain A spike receptor-binding domain, and **(B)** SARS-CoV-2 chain B spike receptor-binding domain relative to their initial structures.

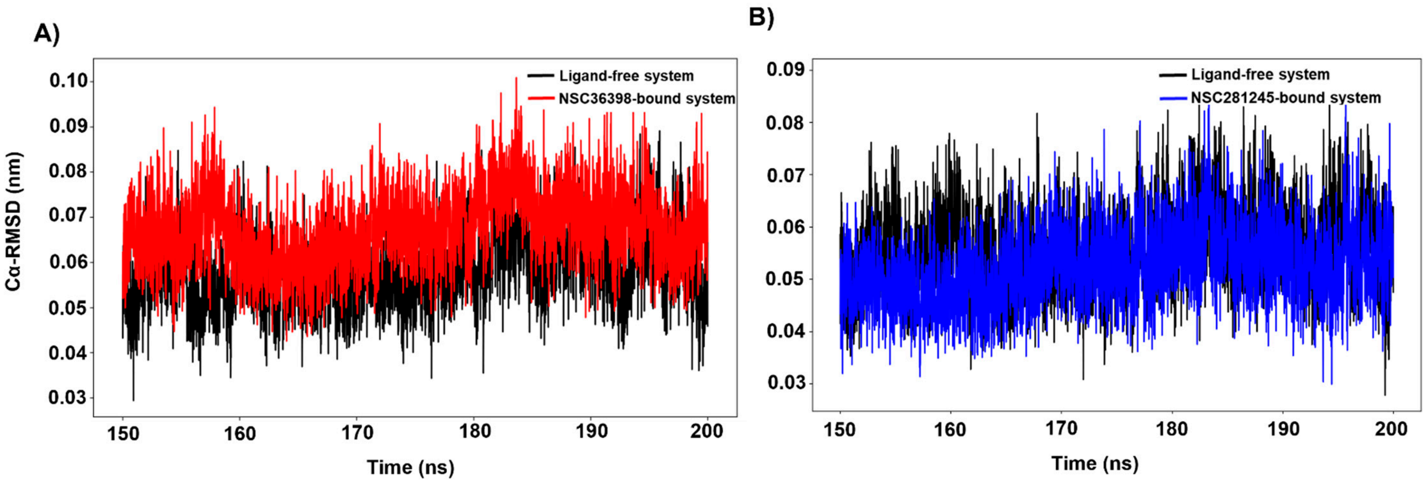

Figure S7: (A) C*α*-RMSF line plot representation of the ligand-free system and NSC281245 binding-pockets **(B)**, and the ligand-free system and NSC36398 binding-pockets. Binding-pocket residues were defined as residues of any atom within 5 Å of the ligand in the initial frame structure.

Table S2: Hydrogen bond donor-hydrogen-acceptor pairs identified between NSC36398 and the protein binding pocket during the 150-200 ns segment of the MD simulation.

H-bond Index	Donor Atom	Hydrogen Atom	Acceptor Atom
0	SER1021OG	SER1021HG	LG31145O3
1	ARG1019NE	ARG1019HE	LG31145O4
2	ARG1019NH2	ARG1019HH21	LG31145O3
3	ARG1019NH2	ARG1019HH21	LG31145O4

4	ASN1023ND2	ASN1023HD21	LG31145O2
5	ARG1019NE	ARG1019HE	LG31145O6
6	ARG1019NH2	ARG1019HH21	LG31145O6
7	ASN1023ND2	ASN1023HD21	LG31145O6
8	LG31145O1	LG31145H6	ASN1023OD1
9	LG31145O1	LG31145H6	ASN1023ND2
10	LG31145O1	LG31145H6	ASN1023ND2
11	LG31145O3	LG31145H8	GLU1017OE1
12	LG31145O3	LG31145H8	GLU1017OE2
13	LG31145O3	LG31145H8	GLU1017O
14	LG31145O3	LG31145H8	ALA1020O
15	LG31145O4	LG31145H9	GLU1017OE1
16	LG31145O4	LG31145H9	GLU1017OE2

Table S3: Hydrogen bond donor-hydrogen-acceptor pairs identified between NSC281245 and the protein binding pocket during the 150-200 ns segment of the MD simulation.

**H-bond Index**	**Donor Atom**	**Hydrogen Atom**	**Acceptor Atom**
0	ARG1019NH1	ARG1019HH11	LG41145O4
1	ARG1019NH2	ARG1019HH21	LG41145O1
2	ARG1019NH2	ARG1019HH21	LG41145O4
3	ARG1019ND2	ARG1019HD21	LG41145O4
4	LG41145O2	LG41145O2	ALA1016O

Table S4: Summarised average energy components and their standard mean errors (SEM) of the NSC36398-bound and NSC281245-bound systems with their respective S2 binding pockets.

**Energy Component**	**NSC36398 ΔEnergy (kcal/mol) ± SEM**	**NSC281245 ΔEnergy (kcal/mol) ± SEM**
$\Delta_{VDWAALS}$	–10.35 ± 0.05	–37.80 ± 0.07
Δ_{EEL}	–9.45 ± 0.08	–12.99 ± 0.13
Δ_{EGB}	14.88 ± 0.06	28.72 ± 0.11
Δ_{ESURF}	–2.15 ± 0.00	–5.56 ± 0.01
Δ_{GGAS}	–19.80 ± 0.08	–50.79 ± 0.14
Δ_{GSOLV}	12.72 ± 0.06	23.16 ± 0.11
$\Delta_{TOTAL/BIND}$	–7.07 ± 0.04	–27.63 ± 0.07

Table S5: Summarised average energy components and their standard mean errors (SEM) of the HSPA8-spike interaction surface (domain level) from the ligand-free, NSC36398-bound and NSC281245-bound systems.

**Energy Component**	**Ligand-free system ΔEnergy (kcal/mol) ± SEM**	**NSC36398 ΔEnergy (kcal/mol) ± SEM**	**NSC281245 ΔEnergy (kcal/mol) ± SEM**
$\Delta_{VDWAALS}$	–106.67 ± 0.53	–81.79 ± 0.39	–107.52 ± 0.49
Δ_{EEL}	–561.05 ± 1.73	–451.05 ± 1.47	–426.03 ± 1.24
Δ_{EGB}	614.11 ± 1.74	508.37 ± 1.29	485.46 ± 1.06
Δ_{ESURF}	–13.84 ± 0.06	–9.92± 0.04	–14.17 ± 0.06
Δ_{GGAS}	–667.72 ± 1.90	–532.84 ± 1.51	–533.56 ± 1.26
Δ_{GSOLV}	600.28 ± 1.70	498.45 ± 1.28	471.30 ± 1.03
$\Delta_{TOTAL/BIND}$	–67.44 ± 0.46	–34.39 ± 0.54	–62.26 ± 0.56

https://doi.org/10.3390/xxxxx
