# Supplementary material for: In-Depth Molecular Dynamics Simulations Reveal Ligand-Induced Modulations of the HSPA8-SARS-CoV-2 Spike Protein Interaction
Source: Int J Mol Sci. 2026 May 12;27(10):4288. doi: 10.3390/ijms27104288 (PMC13207655; doi:10.3390/ijms27104288)
Supplement: Supplementary file 1 [file ijms-27-04288-s001.zip › ijms-3866908-Supplementary_File_S2.pdf]

International Journal of *Molecular Sciences*

MDPI

Supplementary Materials for

In-Depth Molecular Dynamics Simulations Reveal Ligand-Induced Modulations of the HSPA8-SARS-CoV-2 Spike Protein Interaction

Liberty T. Navhaya [1], Mokgerwa Z. Monama [1], Thabe M. Matsebatlela [1] and Xolani H. Makhoba [2,*]

[1] Department of Biochemistry, Microbiology, and Biotechnology, University of Limpopo, Turfloop Campus, Sovenga 7270, South Africa; 202417284@myturf.ul.ac.za (L.T.N.); mokgerwa.monama@ul.ac.za (M.Z.M.); thabe.matsebatlela@ul.ac.za (T.M.M.)

[2] Department of Life and Consumer Sciences, College of Agriculture and Environmental Sciences, University of South Africa (UNISA), Florida Campus, Roodepoort 1709, South Africa

* Correspondence: makhoxh@unisa.ac.za

Note: The material contained herein is supplementary to the article named in the title and published in the International Journal of Molecular Sciences

A. *Energy minimisation parameters utilised in molecular dynamics system setup in both ligand-free and ligand-bound systems*

; minim.mdp - used as input into grompp to generate em.tpr

; Parameters describing what to do, when to stop and what to save

integrator = steep ; Algorithm (steep = steepest descent minimization)

emtol = 1000.0 ; Stop minimization when the maximum force < 1000.0 kJ/mol/nm

emstep = 0.01 ; Minimization step size

nsteps = 50000 ; Maximum number of (minimization) steps to perform

; Parameters describing how to find the neighbors of each atom and how to calculate the interactions

nstlist = 1 ; Frequency to update the neighbor list and long-range forces

cutoff-scheme = Verlet ; Buffered neighbor searching

ns_type = grid ; Method to determine neighbor list (simple, grid)

coulombtype = PME ; Treatment of long-range electrostatic interactions

rcoulomb = 1.0 ; Short-range electrostatic cut-off

rvdw = 1.0 ; Short-range Van der Waals cut-off

pbc = xyz ; Periodic Boundary Conditions in all 3 dimensions

B. *Temperature equilibration parameters utilised in energy equilibration stage of the MD simulation in the ligand-free system*

title = Unbound Protein Complex NVT Equilibration

define = -DPOSRES ; position restrain the protein

; Run parameters

```
integrator              = md            ; leap-frog integrator
nsteps                  = 50000         ; 2 * 50000 = 100 ps
dt                      = 0.002         ; 2 fs
; Output control
nstxout                 = 500           ; save coordinates every 1.0 ps
nstvout                 = 500           ; save velocities every 1.0 ps
nstenergy               = 500           ; save energies every 1.0 ps
nstlog                  = 500           ; update log file every 1.0 ps
; Bond parameters
continuation            = no            ; first dynamics run
constraint_algorithm    = lincs         ; holonomic constraints
constraints             = h-bonds       ; bonds involving H are constrained
lincs_iter              = 1             ; accuracy of LINCS
lincs_order             = 4             ; also related to accuracy
; Nonbonded settings
cutoff-scheme           = Verlet        ; Buffered neighbor searching
ns_type                 = grid          ; search neighboring grid cells
nstlist                 = 10            ; 20 fs, largely irrelevant with Verlet
rcoulomb                = 1.0           ; short-range electrostatic cutoff (in nm)
rvdw                    = 1.0           ; short-range van der Waals cutoff (in nm)
DispCorr                = EnerPres      ; account for cut-off vdW scheme
; Electrostatics
coulombtype             = PME           ; Particle Mesh Ewald for long-range electrostatics
pme_order               = 4             ; cubic interpolation
fourierspacing          = 0.16          ; grid spacing for FFT
; Temperature coupling is on
tcoupl                  = V-rescale                 ; modified Berendsen thermostat
tc-grps                 = Protein Water_and_ions    ; two coupling groups - more accurate
tau_t                   = 0.1     0.1               ; time constant, in ps
ref_t                   = 300     300               ; reference temperature, one for each group, in K
; Pressure coupling is off
pcoupl                  = no            ; no pressure coupling in NVT
; Periodic boundary conditions
pbc                     = xyz           ; 3-D PBC
; Velocity generation
gen_vel                 = yes           ; assign velocities from Maxwell distribution
gen_temp                = 300           ; temperature for Maxwell distribution
gen_seed                = -1            ; generate a random seed
```

C. *Temperature equilibration parameters utilised in energy equilibration stage of the MD simulation in the ligand-bound systems*

```
title                    = Bound Protein Complex NVT Equilibration
define                   = -DPOSRES   ; position restrain the protein
; Run parameters
integrator               = md          ; leap-frog integrator
nsteps                   = 50000       ; 2 * 50000 = 100 ps
dt                       = 0.002       ; 2 fs
; Output control
nstxout                  = 500         ; save coordinates every 1.0 ps
nstvout                  = 500         ; save velocities every 1.0 ps
nstenergy                = 500         ; save energies every 1.0 ps
nstlog                   = 500         ; update log file every 1.0 ps
; Bond parameters
continuation             = no          ; first dynamics run
constraint_algorithm     = lincs       ; holonomic constraints
constraints              = h-bonds     ; bonds involving H are constrained
lincs_iter               = 1           ; accuracy of LINCS
lincs_order              = 4           ; also related to accuracy
; Nonbonded settings
cutoff-scheme            = Verlet      ; Buffered neighbor searching
ns_type                  = grid        ; search neighboring grid cells
nstlist                  = 10          ; 20 fs, largely irrelevant with Verlet
rcoulomb                 = 1.0         ; short-range electrostatic cutoff (in nm)
rvdw                     = 1.0         ; short-range van der Waals cutoff (in nm)
DispCorr                 = EnerPres    ; account for cut-off vdW scheme
; Electrostatics
coulombtype              = PME         ; Particle Mesh Ewald for long-range electro-
statics
pme_order                = 4           ; cubic interpolation
fourierspacing           = 0.16        ; grid spacing for FFT
; Temperature coupling is on
tcoupl                   = V-rescale                  ; modified Berendsen thermostat
tc-grps                  = Protein_Ligand Water_and_ions    ; two coupling groups -
more accurate
tau_t                    = 0.1     0.1                ; time constant, in ps
ref_t                    = 300     300                ; reference temperature, one for
each group, in K
; Pressure coupling is off
pcoupl                   = no          ; no pressure coupling in NVT
; Periodic boundary conditions
pbc                      = xyz         ; 3-D PBC
; Velocity generation
gen_vel                  = yes         ; assign velocities from Maxwell distribution
gen_temp                 = 300         ; temperature for Maxwell distribution
gen_seed                 = -1          ; generate a random seed
```

https://doi.org/10.3390/xxxxx

D. *Pressure equilibration parameters utilised in energy equilibration stage of the MD simulation in the ligand-free system*

```
title                   = Unbound Protein Complex NPT Equilibration
define                  = -DPOSRES   ; position restrain the protein
; Run parameters
integrator              = md         ; leap-frog integrator
nsteps                  = 50000      ; 2 * 50000 = 100 ps
dt                      = 0.002      ; 2 fs
; Output control
nstxout                 = 500        ; save coordinates every 1.0 ps
nstvout                 = 500        ; save velocities every 1.0 ps
nstenergy               = 500        ; save energies every 1.0 ps
nstlog                  = 500        ; update log file every 1.0 ps
; Bond parameters
continuation            = yes        ; Restarting after NVT
constraint_algorithm    = lincs      ; holonomic constraints
constraints             = h-bonds    ; bonds involving H are constrained
lincs_iter              = 1          ; accuracy of LINCS
lincs_order             = 4          ; also related to accuracy
; Nonbonded settings
cutoff-scheme           = Verlet     ; Buffered neighbor searching
ns_type                 = grid       ; search neighboring grid cells
nstlist                 = 10         ; 20 fs, largely irrelevant with Verlet scheme
rcoulomb                = 1.0        ; short-range electrostatic cutoff (in nm)
rvdw                    = 1.0        ; short-range van der Waals cutoff (in nm)
DispCorr                = EnerPres   ; account for cut-off vdW scheme
; Electrostatics
coulombtype             = PME        ; Particle Mesh Ewald for long-range electrostatics
pme_order               = 4          ; cubic interpolation
fourierspacing          = 0.16       ; grid spacing for FFT
; Temperature coupling is on
tcoupl                  = V-rescale               ; modified Berendsen thermostat
tc-grps                 = Protein Water_and_ions  ; two coupling groups - more accurate
tau_t                   = 0.1     0.1             ; time constant, in ps
ref_t                   = 300     300             ; reference temperature, one for each group, in K
; Pressure coupling is on
pcoupl                  = Berendsen               ; Pressure coupling on in NPT
pcoupltype              = isotropic               ; uniform scaling of box vectors
tau_p                   = 2.0                     ; time constant, in ps
ref_p                   = 1.0                     ; reference pressure, in bar
```

compressibility = 4.5e-5 ; isothermal compressibility of water, bar^-1

refcoord_scaling = com

; Periodic boundary conditions

pbc = xyz ; 3-D PBC

; Velocity generation

gen_vel = no ; Velocity generation is off

E. *Pressure equilibration parameters utilised in energy equilibration stage of the MD simulation in the ligand-bound systems*

title = Bound Protein Complex NPT Equilibration

define = -DPOSRES ; position restrain the protein

; Run parameters

integrator = md ; leap-frog integrator

nsteps = 50000 ; 2 * 50000 = 100 ps

dt = 0.002 ; 2 fs

; Output control

nstxout = 500 ; save coordinates every 1.0 ps

nstvout = 500 ; save velocities every 1.0 ps

nstenergy = 500 ; save energies every 1.0 ps

nstlog = 500 ; update log file every 1.0 ps

; Bond parameters

continuation = yes ; Restarting after NVT

constraint_algorithm = lincs ; holonomic constraints

constraints = h-bonds ; bonds involving H are constrained

lincs_iter = 1 ; accuracy of LINCS

lincs_order = 4 ; also related to accuracy

; Nonbonded settings

cutoff-scheme = Verlet ; Buffered neighbor searching

ns_type = grid ; search neighboring grid cells

nstlist = 10 ; 20 fs, largely irrelevant with Verlet scheme

rcoulomb = 1.0 ; short-range electrostatic cutoff (in nm)

rvdw = 1.0 ; short-range van der Waals cutoff (in nm)

DispCorr = EnerPres ; account for cut-off vdW scheme

; Electrostatics

coulombtype = PME ; Particle Mesh Ewald for long-range electrostatics

pme_order = 4 ; cubic interpolation

fourierspacing = 0.16 ; grid spacing for FFT

; Temperature coupling is on

tcoupl = V-rescale ; modified Berendsen thermostat

tc-grps = Protein_Ligand Water_and_ions ; two coupling groups - more accurate

tau_t = 0.1 0.1 ; time constant, in ps

ref_t = 300 300 ; reference temperature, one for each group, in K

; Pressure coupling is on

pcoupl = Berendsen ; Pressure coupling on in NPT

pcoupltype = isotropic ; uniform scaling of box vectors

tau_p = 2.0 ; time constant, in ps

ref_p = 1.0 ; reference pressure, in bar

compressibility = 4.5e-5 ; isothermal compressibility of water, bar^-1

refcoord_scaling = com

; Periodic boundary conditions

pbc = xyz ; 3-D PBC

; Velocity generation

gen_vel = no ; Velocity generation is off

F. *MD production parameters utilised in generating the trajectories in the ligand-free system's MD simulation*

title = Unbound Protein Complex MD Production

; Run parameters

integrator = md ; leap-frog integrator

nsteps = 100000000 ; 2 * 500000 = 1000 ps (1 ns)

dt = 0.002 ; 2 fs

; Output control

nstxout = 0 ; suppress bulky .trr file by specifying

nstvout = 0 ; 0 for output frequency of nstxout,

nstfout = 0 ; nstvout, and nstfout

nstenergy = 5000 ; save energies every 10.0 ps

nstlog = 5000 ; update log file every 10.0 ps

nstxout-compressed = 5000 ; save compressed coordinates every 10.0 ps

compressed-x-grps = System ; save the whole system

; Bond parameters

continuation = yes ; Restarting after NPT

constraint_algorithm = lincs ; holonomic constraints

constraints = h-bonds ; bonds involving H are constrained

lincs_iter = 1 ; accuracy of LINCS

lincs_order = 4 ; also related to accuracy

; Neighborsearching

cutoff-scheme = Verlet ; Buffered neighbor searching

ns_type = grid ; search neighboring grid cells

nstlist = 10 ; 20 fs, largely irrelevant with Verlet scheme

rcoulomb = 1.0 ; short-range electrostatic cutoff (in nm)

rvdw = 1.0 ; short-range van der Waals cutoff (in nm)

; Electrostatics

coulombtype = PME ; Particle Mesh Ewald for long-range electrostatics

pme_order = 4 ; cubic interpolation

fourierspacing = 0.16 ; grid spacing for FFT

; Temperature coupling is on

tcoupl = V-rescale ; modified Berendsen thermostat

tc-grps = Protein Water_and_ions; two coupling groups - more accurate

tau_t = 0.1 0.1 ; time constant, in ps

ref_t = 300 300 ; reference temperature, one for each group, in K

; Pressure coupling is on

pcoupl = Parrinello-Rahman ; Pressure coupling on in NPT

pcoupltype = isotropic ; uniform scaling of box vectors

tau_p = 2.0 ; time constant, in ps

ref_p = 1.0 ; reference pressure, in bar

compressibility = 4.5e-5 ; isothermal compressibility of water, bar^-1

; Periodic boundary conditions

pbc = xyz ; 3-D PBC

; Dispersion correction

DispCorr = EnerPres ; account for cut-off vdW scheme

; Velocity generation

gen_vel = no ; Velocity generation is off

G. *MD production parameters utilised in generating the trajectories in the ligand-bound systems' MD simulation*

title = Bound Protein Complex MD Production

; Run parameters

integrator = md ; leap-frog integrator

nsteps = 100000000 ; 2 * 500000 = 1000 ps (1 ns)

dt = 0.002 ; 2 fs

; Output control

nstxout = 0 ; suppress bulky .trr file by specifying

nstvout = 0 ; 0 for output frequency of nstxout,

nstfout = 0 ; nstvout, and nstfout

nstenergy = 5000 ; save energies every 10.0 ps

nstlog = 5000 ; update log file every 10.0 ps

nstxout-compressed = 5000 ; save compressed coordinates every 10.0 ps

compressed-x-grps = System ; save the whole system

; Bond parameters

continuation = yes ; Restarting after NPT

constraint_algorithm = lincs ; holonomic constraints

constraints = h-bonds ; bonds involving H are constrained

lincs_iter = 1 ; accuracy of LINCS

```
lincs_order              = 4          ; also related to accuracy
; Neighborsearching
cutoff-scheme            = Verlet     ; Buffered neighbor searching
ns_type                  = grid       ; search neighboring grid cells
nstlist                  = 10         ; 20 fs, largely irrelevant with Verlet scheme
rcoulomb                 = 1.0        ; short-range electrostatic cutoff (in nm)
rvdw                     = 1.0        ; short-range van der Waals cutoff (in nm)
; Electrostatics
coulombtype              = PME        ; Particle Mesh Ewald for long-range electrostat-
ics
pme_order                = 4          ; cubic interpolation
fourierspacing           = 0.16       ; grid spacing for FFT
; Temperature coupling is on
tcoupl                   = V-rescale              ; modified Berendsen thermostat
tc-grps                  = Protein_Ligand Water_and_ions; two coupling groups - more
accurate
tau_t                    = 0.1     0.1            ; time constant, in ps
ref_t                    = 300     300            ; reference temperature, one for each
group, in K
; Pressure coupling is on
pcoupl                   = Parrinello-Rahman      ; Pressure coupling on in NPT
pcoupltype               = isotropic              ; uniform scaling of box vectors
tau_p                    = 2.0                    ; time constant, in ps
ref_p                    = 1.0                    ; reference pressure, in bar
compressibility          = 4.5e-5                 ; isothermal compressibility of water,
bar^-1
; Periodic boundary conditions
pbc                      = xyz        ; 3-D PBC
; Dispersion correction
DispCorr                 = EnerPres   ; account for cut-off vdW scheme
; Velocity generation
gen_vel                  = no         ; Velocity generation is off
```

H. *Block averaging script utilised in analysing the ligand-free and ligand-bound systems' convergence*

```
import pandas as pd
import matplotlib.pyplot as plt
import argparse
import statistics as stat
import math


parser = argparse.ArgumentParser()
parser.add_argument("--input_filename", type=str, help="Comma delimmited .txt or .csv
filename in a \'frames,value\' layout with no headers.")
argv = parser.parse_args()
```

```
print("Reading data...\n")
# Read in the datas
df = pd.read_csv(argv.input_filename, header=None)

# Captures the values and timeframes
sample = list(df.iloc[:,1].values)
sample = sample[0:]
sample_frames = list(df.iloc[:,0].values)
sample_frames = sample_frames[0:]

# Captures the number of frames
n_frames = len(sample)

# Sets block sizes
block_sizes = list(range(1,n_frames + 1))

def make_data_dict(sample,block_sizes):
    data_dict = {}

    print("Creating blocked data...\n")
    for block_size in block_sizes:
        for i in range(0, len(sample), block_size):
            if f"{block_size}" in data_dict:
                data_dict[f"{block_size}"] += [sample[i:i+block_size]]
            else:
                data_dict[f"{block_size}"] = [sample[i:i+block_size]]

    print("Cleaning blocked data...\n")
    clean_data_dict = {}
    for key,value in data_dict.items():
        data = []
        for item in value:
            if len(item) == int(key):
                data.append(item)
        clean_data_dict[key] = data

    return clean_data_dict

def calc_plot_sem():


    data_dict = make_data_dict(sample,block_sizes)
```

```
    block_sems = []
    block_sems_str = ""

    print("Calculating SEM...\n")
    for key,data in data_dict.items():
        block_size = int(key)
        n_blocks = len(data)

        # Calculates the standard mean error for data with block size of 1
        if block_size == 1:
            data = [val[0] for val in data]

            block_std_err_mean = (stat.stdev(data) / math.sqrt(n_blocks))
        # Calculates the standard mean error for data with 1 block
        elif n_blocks == 1:
            data = data[0]
            block_std_err_mean = (stat.stdev(data) / math.sqrt(n_blocks))
        # Calculates the standard mean error for data with several blocks
        else:
            block_avgs = []
            for values in data:
                block_avg = stat.mean(values)
                block_avgs.append(block_avg)
            block_std_err_mean = (stat.stdev(block_avgs) / math.sqrt(n_blocks))

        # Captures the standard error of mean per block size
        block_sems.append(block_std_err_mean)
        block_sems_str += f"{block_std_err_mean}\n"

    fig, ax = plt.subplots()
    # Plots values with SEM error bars against block size
    ax.errorbar(block_sizes,sample[0:len(block_sizes)], yerr=block_sems,fmt="--",lin-
ewidth=0.5, alpha=0.5,ecolor="red",capsize=4)
    ax.plot(block_sizes,sample[0:len(block_sizes)], color = "black")
    ax.set_xlabel("Block sizes")
    ax.set_ylabel("RMSD (nm)")
    plt.savefig("Data_SEM_BlockSize_{}.png".format(block_size), format="png",
dpi=600)
    print(f"Wrote: Data_SEM_BlockSize_{block_size}.png\n")

    fig, ax = plt.subplots()
    # Plots the standard error of block average means against block size
    ax.plot(block_sizes,block_sems, color = "black")
    ax.set_xlabel("Block sizes")
```

```
    ax.set_ylabel("SEM")
    plt.savefig("SEM_BlockSize_{}.png".format(block_size), format="png", dpi=600)
    print(f"Wrote: SEM_BlockSize_{block_size}.png\n")

    # Captures the calculated SEM values
    write_file = open("SEM_{}.csv".format(block_size),"w")
    write_file.writelines(block_sems_str)
    write_file.close()

calc_plot_sem()
```

I. *Mann–Whitney U test script utilised determining the significant structural effects of ligand binding to the protein structures in the ligand-bound systems compared to the ligand-free system*

```
import numpy as np
import pandas as pd
from scipy.stats import mannwhitneyu
import matplotlib.pyplot as plt

# Config
input_file = "file.csv"
af = pd.read_csv(input_file)
LIGAND_FREE = "Ligand-free system"
BOUND_LIST = ["NSC36398-bound system", "NSC281245-bound system"]  # list of bound columns to compare

# Helper functions
def integrated_autocorr_time(x, max_lag=5000):
    x = np.asarray(x) - np.mean(x)
    n = len(x)
    if n < 2:
        return 0.0
    f = np.fft.rfft(x, n=2*n)
    acov = np.fft.irfft(f * np.conjugate(f))[:n] / n
    acorr = acov / acov[0]
    tau = 0.5
    for t in range(1, min(n, max_lag)):
        if acorr[t] <= 0:
            break
        tau += acorr[t]
    return tau

def cliffs_delta(a, b):
    a, b = np.asarray(a), np.asarray(b)
```

```
    comp = np.sign(a[:, None] - b[None, :])
    return comp.sum() / (len(a) * len(b))

def permutation_mannwhitney_p(x, y, n_perms=20000, seed=42):
    rng = np.random.default_rng(seed)
    combined = np.concatenate([x, y])
    n1 = len(x)
    u_obs, _ = mannwhitneyu(x, y, alternative='two-sided')
    center = n1 * len(y) / 2.0
    count = 0
    for _ in range(n_perms):
        rng.shuffle(combined)
        u_perm, _ = mannwhitneyu(combined[:n1], combined[n1:], alternative='two-
sided')
        if abs(u_perm - center) >= abs(u_obs - center):
            count += 1
    return (count + 1) / (n_perms + 1), u_obs

def bootstrap_CI_two_sample(xs, ys, statfunc, n_boot=3000, seed=0):
    rng = np.random.default_rng(seed)
    boot = np.empty(n_boot)
    n1, n2 = len(xs), len(ys)
    for i in range(n_boot):
        bx = rng.choice(xs, size=n1, replace=True)
        by = rng.choice(ys, size=n2, replace=True)
        boot[i] = statfunc(bx, by)
    return np.percentile(boot, [2.5,97.5])

# Clean input
# drop Time if present
if 'Time' in af.columns:
    af = af.drop(columns=['Time'])

# Core compare function (call for each bound ligand)
def compare_groups(af, colA, colB, n_perms=20000, n_boot=3000, plot=True):
    if colA not in af.columns or colB not in af.columns:
        raise KeyError("Columns not found. Available: " + ", ".join(af.columns.tolist()))
    x_all = pd.to_numeric(af[colA], errors='coerce').dropna().values
    y_all = pd.to_numeric(af[colB], errors='coerce').dropna().values

    # estimate autocorr time and choose conservative spacing = ceil(2*tau)
    tau_x = integrated_autocorr_time(x_all)
    tau_y = integrated_autocorr_time(y_all)
```

```
    spacing = max(1, int(np.ceil(2 * tau_x)), int(np.ceil(2 * tau_y)))
    x_sub = x_all[::spacing]
    y_sub = y_all[::spacing]

    # medians & IQR
    med_x = np.median(x_sub); med_y = np.median(y_sub)
    iqr_x = np.percentile(x_sub, [25,75]); iqr_y = np.percentile(y_sub, [25,75])

    # Mann-Whitney (exact if available)
    try:
        u_stat, p_val = mannwhitneyu(x_sub, y_sub, alternative='two-sided',
method='exact')
        method = 'exact'
    except TypeError:
        p_val, u_stat = permutation_mannwhitney_p(x_sub, y_sub, n_perms=n_perms)
        method = f'permutation (n={n_perms})'

    # Cliff's delta and bootstrap CI
    cd = cliffs_delta(x_sub, y_sub)
    cd_lo, cd_hi = bootstrap_CI_two_sample(x_sub, y_sub, cliffs_delta, n_boot=n_boot,
seed=1)
    md_lo, md_hi = bootstrap_CI_two_sample(x_sub, y_sub, lambda a,b: np.median(a)-
np.median(b), n_boot=n_boot, seed=2)

    # print result
    print("\n--- Comparison:", colA, "vs", colB, "---")
    print(f"tau_int: {colA}={tau_x:.2f}, {colB}={tau_y:.2f}; spacing = {spacing}")
    print(f"n (orig): {len(x_all)}, {len(y_all)}; n (subsampled): {len(x_sub)}, {len(y_sub)}")
    print(f"Medians (IQR): {colA} = {med_x:.6f} ({iqr_x[0]:.6f}-{iqr_x[1]:.6f}); {colB} =
{med_y:.6f} ({iqr_y[0]:.6f}-{iqr_y[1]:.6f})")
    print(f"Mann-Whitney ({method}): U = {u_stat:.2f}, p = {p_val:.6g}")
    print(f"Cliff's delta = {cd:.3f}, 95% CI = [{cd_lo:.3f}, {cd_hi:.3f}]")
    print(f"Median diff (A - B) = {med_x-med_y:.6f}, 95% CI = [{md_lo:.6f}, {md_hi:.6f}]")

    if plot:
        plt.figure(figsize=(7,3.5))
        plt.boxplot([x_sub, y_sub], labels=[colA, colB], vert=False)
        plt.scatter(x_sub, np.repeat(1, len(x_sub)), marker='o')
        plt.scatter(y_sub, np.repeat(2, len(y_sub)), marker='o')
        plt.xlabel("RMSD (nm)")
        plt.title(f"Subsampled RMSD - {colA} vs {colB}")
        plt.tight_layout()
        plt.show()
```

```
    # return dictionary for programmatic use
    return {
        'tau': (tau_x, tau_y), 'spacing': spacing,
        'n_sub': (len(x_sub), len(y_sub)), 'median': (med_x, med_y),
        'IQR': (iqr_x, iqr_y), 'U': u_stat, 'p': p_val, 'cliff': cd,
        'cliff_CI': (cd_lo, cd_hi), 'median_diff_CI': (md_lo, md_hi)
    }

# Run comparisons (loop over bound ligands)
for bound_col in BOUND_LIST:
    compare_groups(af, LIGAND_FREE, bound_col, n_perms=20000, n_boot=3000,
plot=True)
```
